# Enhancing tetraphenylethene cyclization as photoswitch

**DOI:** 10.1002/smo.20230003

**Published:** 2023-08-06

**Authors:** Yue Wu, Yiran Ren, Xiaoxuan Zeng, Honglong Hu, Mengqi Li, Junzi Li, Tingchao He, Xin‐Shun Li, Zhen‐Qiang Yu, Wei‐Hong Zhu

**Affiliations:** ^1^ College of Chemistry and Environmental Engineering Shenzhen University Shenzhen China; ^2^ Laboratory for Advanced Materials and Joint International Research Laboratory of Precision Chemistry and Molecular Engineering Feringa Nobel Prize Scientist Joint Research Center Shanghai Key Laboratory of Functional Materials Chemistry Institute of Fine Chemicals Frontiers Science Center for Materiobiology and Dynamic Chemistry School of Chemistry and Molecular Engineering East China University of Science and Technology Shanghai China; ^3^ Key Laboratory of Optoelectronic Devices and Systems of Ministry of Education and Guangdong Province College of Physics and Optoelectronic Engineering Shenzhen University Shenzhen China

**Keywords:** aggregation‐induced emission, diarylethene, photocyclization, photoswitch, tetraphenylethene

## Abstract

Tetraphenylethene (TPE), a star building block with promising aggregation‐induced emission, has received much interest. Given that its intramolecular Woodward‐Hoffmann cyclic intermediate instantaneously converts back to the original state within several picoseconds, the essentially photochromic characteristic of TPE is little investigated. Achieving a visible photocyclization of TPE is still an unsolved issue and considered as the bottleneck in the further advancement of applications. We report a strategy of attaching carbonate ester onto the TPE skeleton (TPE‐4C) to enhance TPE photocyclization stability. As demonstrated, the incorporated cholesteryloxycarbonyloxy substituents in TPE‐4C can increase the energy barrier for cycloreversion, thereby exhibiting extremely thermal stability of photocyclic intermediate upon UV irradiation, prolonging its lifetime from 63 picoseconds to 46 s by 7.2 × 10^11^‐fold. The photoinduced cyclization of TPE‐4C could be monitored with naked eyes, and the photocyclization/cycloreversion is achieved by turning on/off UV light along with a relative fatigue resistance. Encapsulation of TPE‐4C into the liquid crystal can induce a striking phase transformation (achiral↔chiral), which can be applicable to encode optical information. Employing carbonate ester into the TPE unit plays a vital role in enhancing the unprecedented TPE photocyclization stability, providing a toolbox to allow TPE‐based photocyclization to be visually monitored.

## Graphical abstract

Tetraphenylethene (TPE), a molecule with promising aggregation‐induced emission, has a photochromic diarylethene structure. We have solved the issue of whether diarylethene‐featured TPE derivatives bestow photo‐switchable characteristics, and successfully demonstrated a visual and reversible photocyclization of TPE by attaching rigid liquid crystal mesogen cholesterols onto the four benzene rings of TPE by decelerating the intramolecular rotations.

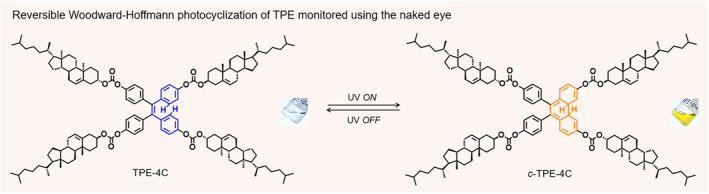

## 1 INTRODUCTION

Since first reported by Tang and coworkers in 2007,^[1]^ tetraphenylethene (TPE) has promoted intense interest in the development of aggregation‐induced emission (AIE)[^2^, ^3^, ^4^, ^5^, ^6^, ^7^, ^8^] applications such as theranostics,[^9^, ^10^, ^11^] chiral applications,[^12^, ^13^, ^14^, ^15^, ^16^] and also in the assembly of more complex structures.[^17^, ^18^, ^19^] Therefore, TPE is well regarded as a star AIE building block to date. Revealing the dynamic stereostructure change of TPE is crucial to the discovery and understanding of its specific physical phenomena.[^20^, ^21^] Moreover, in the propeller‐like conformation, the molecular structure of TPE clearly resembles a typical photochromic diarylethene (DAE) characteristic.[^22^, ^23^, ^24^, ^25^, ^26^, ^27^, ^28^, ^29^, ^30^, ^31^, ^32^, ^33^] This then poses one question on whether DAE‐featured TPE or TPE derivatives displays reversible photoswitching by obeying the Woodward‐Hoffmann rearrangement rule?

However, rare visible photochromism of TPE or TPE derivatives has been reported due to the extremely low switchable conversion.[^34^, ^35^] According to the Hückel rule, the strong aromaticity of the photochromic structure can greatly enhance the ground‐state energy difference between the open and closed forms, ultimately causing the thermal instability of the cyclization form.[^36^, ^37^] Compared to other polar ethene bridges, such as perfluorocyclopentene,[^38^, ^39^] maleic imide,[^40^, ^41^] or benzobisthiadiazole,[^42^, ^43^, ^44^, ^45^] the strong aromaticity of TPE unit immensely restricts its thermal stability of closed form. Hence, how to establish a visible photocyclization of TPE is still an unsolved issue and considered as the bottleneck in the further advancement of TPE‐based application.

**FIGURE 1:**
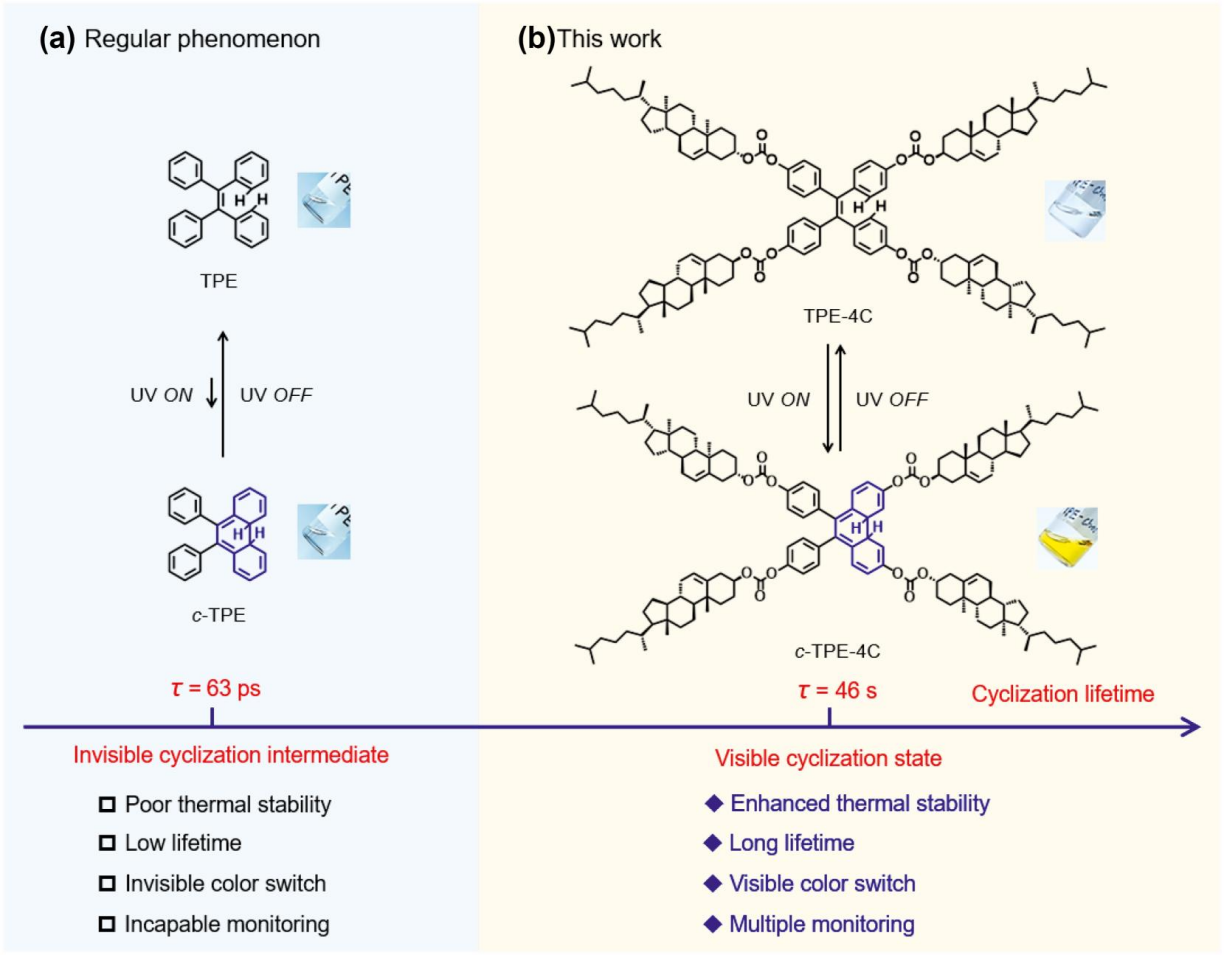
Strategy with the incorporation of cholesteric substituents into TPE to achieve reversible visible photocyclization/cycloreversion. Upon irradiation with UV light, diarylethene structures TPE (a) are incapable of visible photocyclization whereas TPC‐4C (b), with cholesteryloxycarbonyloxy substituents, does display photocyclization monitored by the naked eyes. DAE, diarylethene; TPE, tetraphenylethene.

Given the strong intrinsic aromaticity of the TPE building block, we attempted to make use of the substituent strategy to enhance the photocyclization stability for visible photoswitching (Figure 1). Four cholesterols, a type of rigid liquid crystal (LC) mesogen, were attached to the TPE building block via carbonate linkers to form TPE‐4C. Unexpectedly, upon UV irradiation, TPE‐4C displayed visible photocyclization with a reversible transformation between colorless (TPE‐4C) and yellow (*c*‐TPE‐4C). Here, we conclude that the incorporated cholesteryloxycarbonyloxy units could increase the cycloreversion energy barrier, and obviously enhance the thermal stability of the Woodward–Hoffmann cyclic *c*‐TPE‐4C. Furthermore, taking advantage of chiral liquid crystallinity of cholesterol, we also encapsulated TPE‐4C into an achiral LC matrix, 4′‐pentyl‐4‐biphenylcarbonitrile (5CB, Figure S1), to produce an LC co‐assembly system, TPE‐4C⊂5CB. Interestingly, TPE‐4C⊂5CB exhibited a dramatic photoswitching between the achiral (nematic) and chiral (cholesteric) phases upon UV irradiation. The dynamic photocyclization of a TPE derivative with carbonate ester can thus be visibly monitored from multiple perspectives, such as color, absorption, and assembly.

## 2 RESULTS AND DISCUSSION

### 2.1 Ultrafast dynamic stereostructure changes of TPE from photocyclization to cycloreversion within picoseconds

**FIGURE 2:**
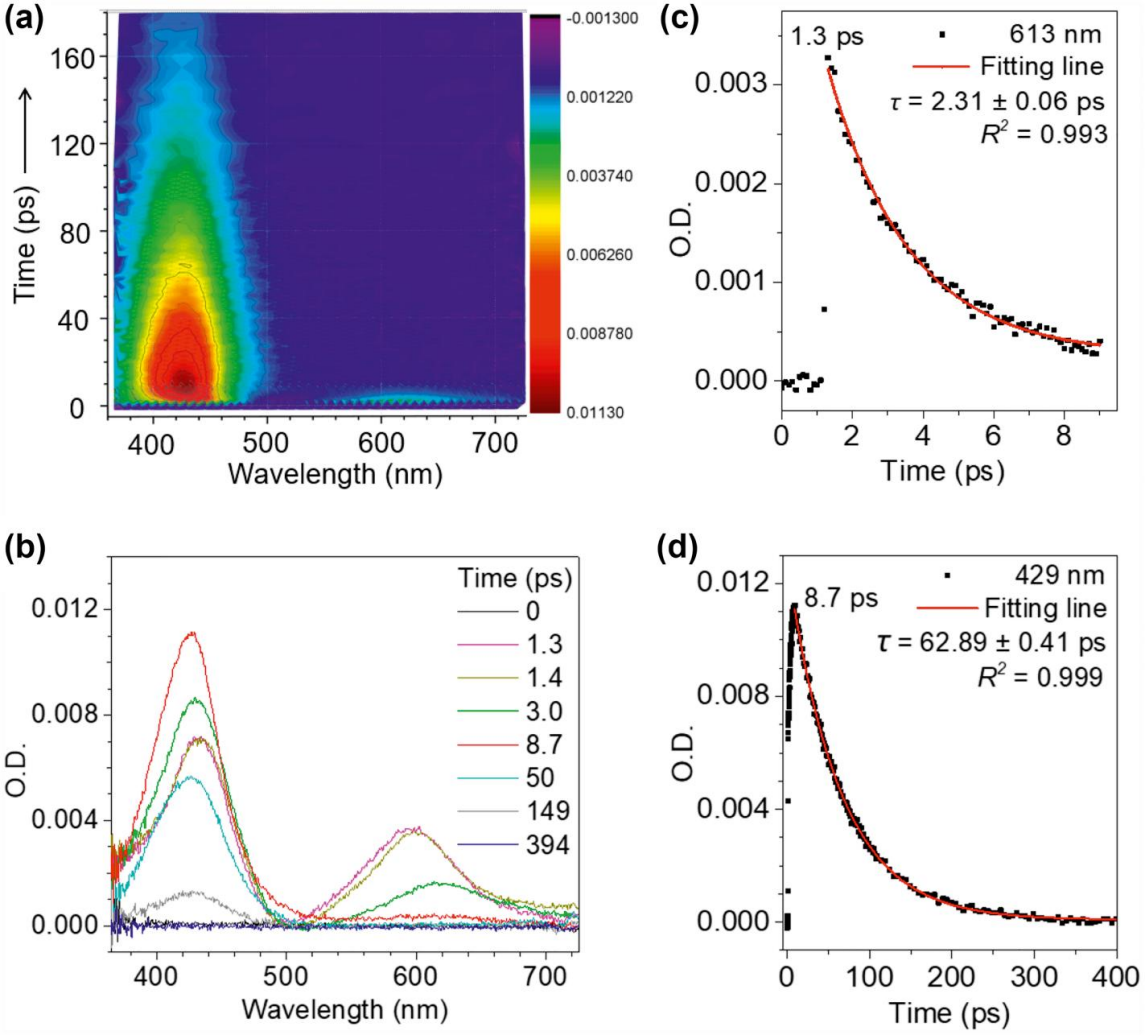
Ultrafast dynamic stereostructure changes of TPE from *cis‐trans* isomerization to photocyclization, and then to cycloreversion within picoseconds. (a) Evolution of 2D ultrafast absorption spectra in dilute THF solution (10 mM) upon excitation with a flash laser of 350 nm (40 mW cm^−2^) under air environment. (b) Transient absorption spectra of TPE monitored at 0, 1.3, 1.4, 3.0, 8.7, 50, 149, and 394 ps, respectively. Absorption decay of TPE solution at 613 nm (c) or 429 nm (d). THF, tetrahydrofuran; TPE, tetraphenylethene.

The photocyclization of TPE is not visually observed by the naked eyes, and no change in regular absorption spectra can be monitored. Thus, we employed the ultrafast transient absorption spectroscopy to observe the dynamic behavior of TPE. Figure 2 displays the evolution of ultrafast transient absorption spectra of TPE in a dilute tetrahydrofuran (THF) solution upon excitation with a flash laser of 350 nm. Firstly, the twisting process of the central C=C bond is reflected in the excited state absorption spectra shown in Figure 2b,c and Figure S2 in Supporting Information. The build‐up of the band at 596 nm (1.3 ps) with its red‐shift to 613 nm is attributed to the central C=C bond elongation associated with the quasi C=C bond twisting. On the picosecond timescale between 1.3 and 8.7 ps, sequential depopulation of the band at 613 nm is attributed to the quasi C=C bond twisting, indicating that the transients in the emissive state revert to the shortening of the elongated C=C bond took place with a 2.3 ps of lifetime.^[46]^ Besides, we conducted a theoretical prediction of TPE. As illustrated in Figure S3 and Table S1, the central C=C double bond reduces its bond order, and akin to the photoexcited ethylene molecule acquires a charge‐resonance (*S*_1_ min) character. The excited state dynamics follows a twisting motion around the central bond until the conical intersection (CI) with a ground state is met. Thus, the *cis‐trans* isomerization along the central C=C can take place, which is in accordance with the abovementioned experiment results.^[47]^

Accompanying the absorption intensity decrease at 613 nm, the growing absorption intensity at 429 nm could be attributed to the emerging of a new compound‐cyclic *c*‐TPE.[^46^, ^47^] It demonstrates that the dominate process is converted from central C=C bond twisting to photocyclization, and a new transient absorption peak appeared at 429 nm upon the UV flash laser excitation (Figure 2a,b), reaching maxima within almost 8.7 ps. Upon the fast absorption diminishment, the thermal decay lifetime was fitted as 63 ps. After 394 ps, its intensity dropped by nearly 100% (Figure 2d). Here, the observed transient absorption peak can be ascribed to an ultrafast photo‐induced Woodward‐Hoffmann cyclic intermediate *c*‐TPE (Figure 1a),[^46^, ^47^] whereas the cyclic intermediate was quickly converted back to the open form TPE within picoseconds, indicative of the extremely thermal instability of *c*‐TPE.

### 2.2 Realization of visible photocyclization of TPE with incorporation of cholesteryloxycarbonyloxy substituents

**FIGURE 3:**
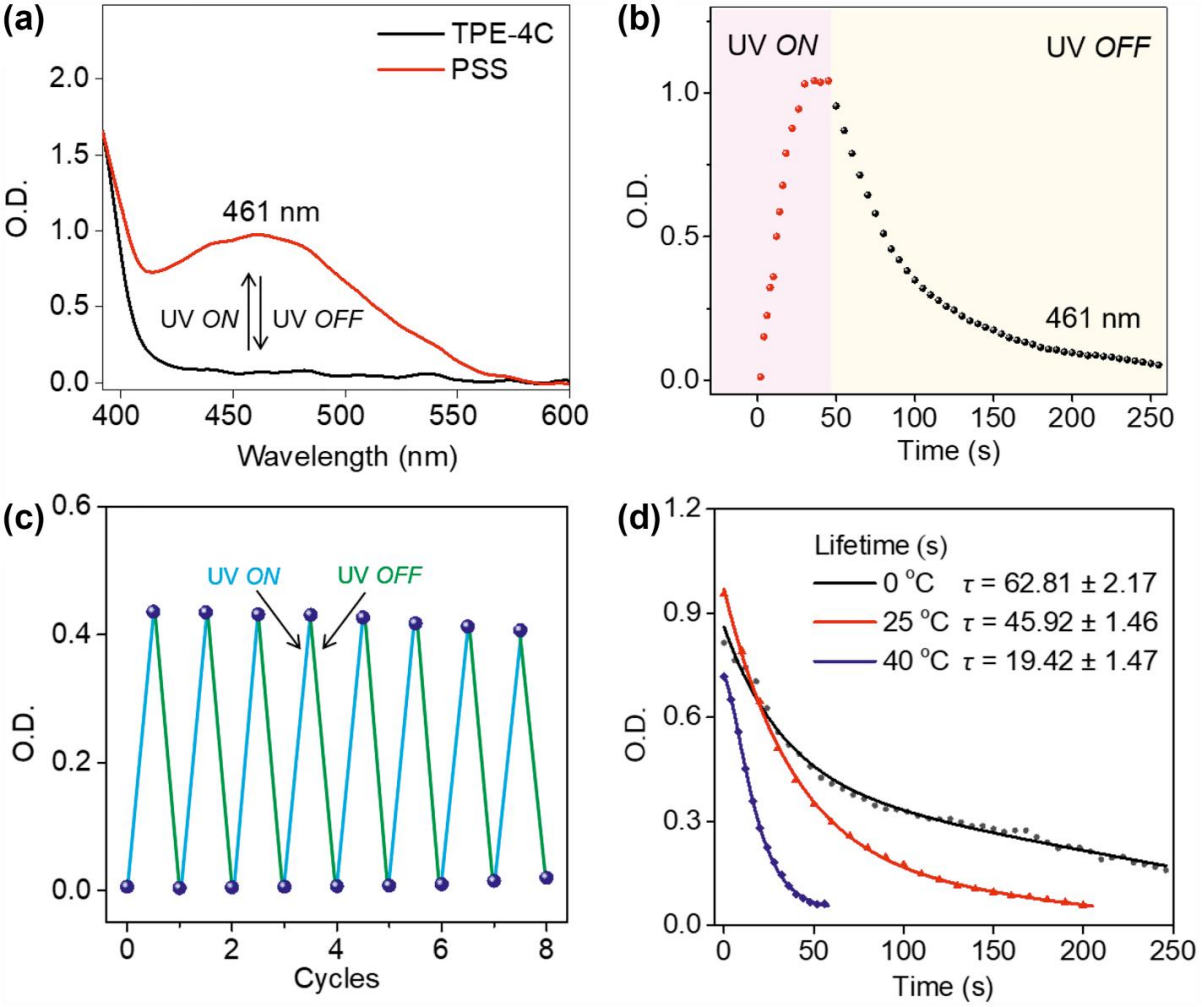
Reversible photocyclization/cycloreversion of TPE‐4C. (a) Photocyclization from TPE‐4C to PSS (1.0 ⨯ 10^−2^ M, THF) upon irradiation at 365 nm and cycloreversion from PSS to ring‐open form on switching off the light source. (b) Photocyclization and cycloreversion kinetics measured at 461 nm as a function of UV on and off time. (c) On‐off toggling of photoswitch between ring‐open and ring‐closed forms by alternating between UV (365 nm, 100 mW cm^−2^) on and off in an air environment, demonstrating a relative fatigue resistance and reversibility. (d) Dynamic decay curves of cycloreversion from PSS to TPE‐4C (10 mM, THF) under air when switching off 365 nm light source at 0, 25, and 40°C, respectively. PSS, photostationary state; THF, tetrahydrofuran.

Next, we focused on the reversible photocyclization of TPE‐4C containing four rigid cholesterol substituents. In contrast with TPE, colorless TPE‐4C, which absorbs “*zero*” in the visible region, can be photoconverted to the yellow ring‐closed form, *c*‐TPE‐4C, upon irradiation at 365 nm (Figures 1b and 3a). Due to the increasing conjugation effect, the ring‐closed form has an absorption peak at 461 nm, and the appearance of the yellow‐complement band is a characteristic indicator of a ring‐open to ring‐closed transformation. Noticeably, only two benzene rings on one side formed cyclization, and other‐side two benzene rings did not due to the molecular rearrangement. Impressively, the photostationary state (PSS) was reached within 30 s following irradiation at 365 nm (100 mW cm^−2^; concentration: 10 mM), indicative of a fast response to light (Figure 3b). When the source of UV irradiation was removed, the photochemically generated *c*‐TPE‐4C could thermally convert back to the original colorless ring‐open form, TPE‐4C.

Repeatedly toggling the molecular photoswitch on and off between ring‐open TPE‐4C and ring‐closed *c*‐TPE‐4C showed that the system has a relative fatigue resistance (Figure 3c) and confirmed that TPE‐4C photocyclization is reversible, unlike one‐time dehydrogenative photocyclization. Next, we conducted the reversible photoswitching experiment in N_2_ environment (Figure S4). The good fatigue resistance and reversibility of TPE‐4C were demonstrated by almost invariable absorption by monitoring at least 40 switching cycles upon alternating UV *on* and *off* irradiation. Different reversion efficiencies were observed when the PSS multiwfn (*c*‐TPE‐4C) was thermally converted back to ring‐open TPE‐4C at different temperatures (Figure 3d); the rate constants were 1.06 ⨯ 10^−2^, 2.02 ⨯ 10^−2^, and 4.30 ⨯ 10^−2^ s^−1^ at 0, 25, and 40°C, corresponding to the fitted lifetime of *c*‐TPE‐4C were 62.81, 45.92, and 19.42 s, respectively. Compared to the lifetime of *c*‐TPE (*τ* = 63 ps) at 25°C, the lifetime of the Woodward‐Hoffmann cyclic *c*‐TPE‐4C (*τ* = 45.92 s) was prolonged by 7.2 × 10^11^‐fold. This result demonstrates that the incorporated cholesteryloxycarbonyloxy substituent strategy could remarkably decelerate the cycloreversion reaction rate, thereby obviously enhancing the thermal stability of cyclization state.

For better demonstrating the visible photocyclization rationale of TPE‐4C, four methoxy, carboxy, t‐butyl, and triphenylamine groups were attached to the TPE skeleton to form TPE‐4OMe (oxygen atom and electron donor), TPE‐4A (electron acceptor), TPE‐4Bu (steric hindrance), and TPE‐4TPA (huge substituent and electron donor), respectively (Figure S5). Similar to TPE, their photocyclizations are not visually observed by the naked eyes, and there is no change in the absorption spectra (Figure S6). Next, we attached four propoxycarbonyloxy groups onto TPE moiety to obtain TPE‐4P (Figure S5). Interestingly, TPE‐4P can be photoconverted to the yellow ring closed form, *c*‐TPE‐4P, upon irradiation at 365 nm (Figure S6e,f). The ring‐closed form has an absorption peak at 463 nm, and the appearance of the yellow‐complement band is a characteristic indicator of a ring‐open to ring‐closed transformation. The PSS was reached within 60 s at 365 nm (100 mW cm^−2^, concentration: 10 mM). When the UV irradiation was removed, the photochemically generated *c*‐TPE‐4P was converted back to the original colorless ring‐open form, TPE‐4P. Thus, the visible photoswitching of TPE‐4C and TPE‐4P proves that the carbonate ester is a pivotal factor contributing to the visible photocyclization of TPE skeleton.

**FIGURE 4:**
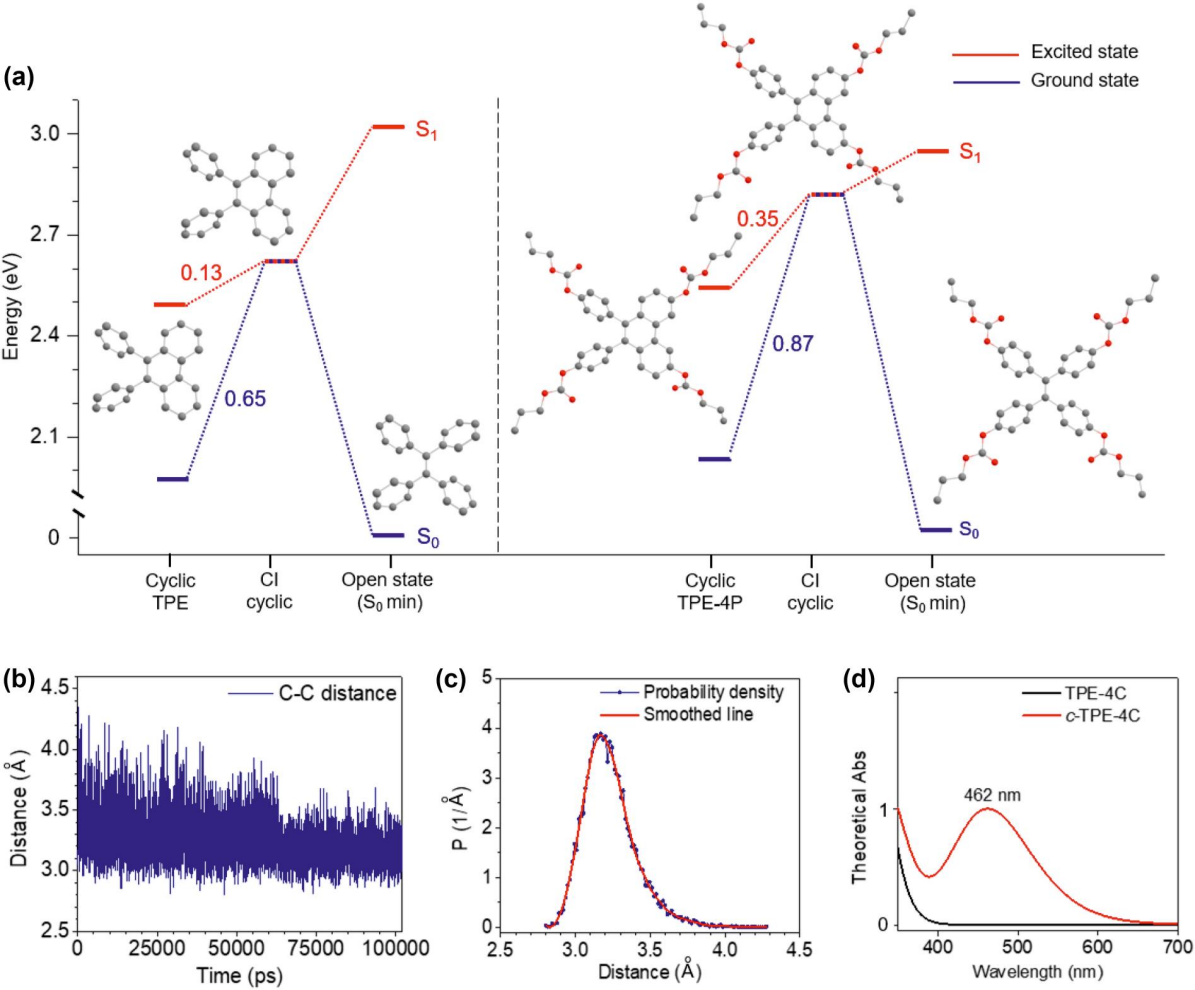
Theoretical calculations for photocyclization of TPE‐4C. (a) Computed energy profiles of TPE and TPE‐4P in THF solution. The Woodward–Hoffmann cyclic states (*c*‐TPE and *c*‐TPE‐4P) can thermally convert back to the ground states of open forms (TPE and TPE‐4P). (b) Dynamic distance evolution between reactive carbon atoms in *anti*‐parallel TPE‐4C (THF). (c) Statistics of dynamic distance between reactive carbons in *anti*‐parallel TPE‐4C. (d) Calculated absorption spectra of ring‐open form (TPE‐4C, black line) and ring‐closed form (*c*‐TPE‐4C, red line) in THF. THF, tetrahydrofuran; TPE, tetraphenylethene.

We also performed the theoretical calculation to further study the visible photoswitching mechanism of carbonate‐ester‐based TPE (Figure 4a and Table S1). Given the huge number of TPE‐4C atoms (346 atoms), we thus used TPE‐4P (98 atoms) as a model for the theoretical calculation in this part. The cycloreversion process of TPE and TPE‐4P molecules was studied by spin‐flip time‐dependent density functional theory (SF‐TDDFT) method. The optimized *S*_0_ state of the open state was set as the ground state (0 eV). Due to the thermal dynamic, the cyclic state dynamics follows cycloreversion between two phenyl rings, and the CI cyclic is first met. This CI state can be quickly restored to the original ground state. By studying their barriers between the cyclic state and CI cyclic state, we found that *c*‐TPE‐4P attaching carbonate esters (Δ*S*_1_ = 0.35 eV, Δ*S*_0_ = 0.87 eV) should overcome a higher barrier than *c*‐TPE (Δ*S*_1_ = 0.13 eV, Δ*S*_0_ = 0.65 eV), resulting in a slower cycloreversion reaction rate than *c*‐TPE. These results further prove that the carbonate ester group plays a key role in the enhanced thermal stability of the TPE‐based cyclic state.

TPE‐4C photocyclization is a type of photochromic phenomenon of DAEs but according to an earlier report, one prerequisite that needs to be satisfied is that the distance between the two reactive carbon atoms in the *anti*‐parallel configuration should be less than 4.2 Å.^[48]^ Given the unique TPE‐characteristic windmilling (intramolecular rotations),[^49^, ^50^] all TPE‐4C molecules can realize photocyclization in theory. We therefore carried out molecular dynamic simulation to investigate the two reactive carbon atoms. Long‐range electrostatic interactions were accounted for using the Ewald method, and the parameters for each like‐site interaction were obtained using the OPLSS‐AA force field.^[51]^ Atomic coordinate statistics between two benzene rings were calculated. As shown in Figure 4b,c, the distance between the reactive carbon atoms (*anti‐*parallel TPE‐4C in THF) almost always lies between 2.8 and 4.0 Å and the average distance is 3.3 Å, thus meeting the prerequisite for photocyclization. Besides, the absorption spectra of ring‐open and ‐closed TPE‐4C were also calculated using time‐dependent density functional theory (Figure 4d and Figures S7 and S8). The calculated absorption spectrum of *c*‐TPE‐4C was in good agreement with the experimentally determined spectrum (Figure 3a) in the range of 400–500 nm, further confirming the photocyclization by TPE‐4C. Due to the increasing conjugation effect, a new band around 462 nm emerges. Specially, the calculated maximal absorption wavelength (462 nm) fits the measured peak wavelength (461 nm) very well. Both the simulation of the reactive carbon atoms and the absorption spectra indirectly confirmed the possibility of photocyclization by TPE‐4C.

### 2.3 Encoding optical information in LC assembly

**FIGURE 5:**
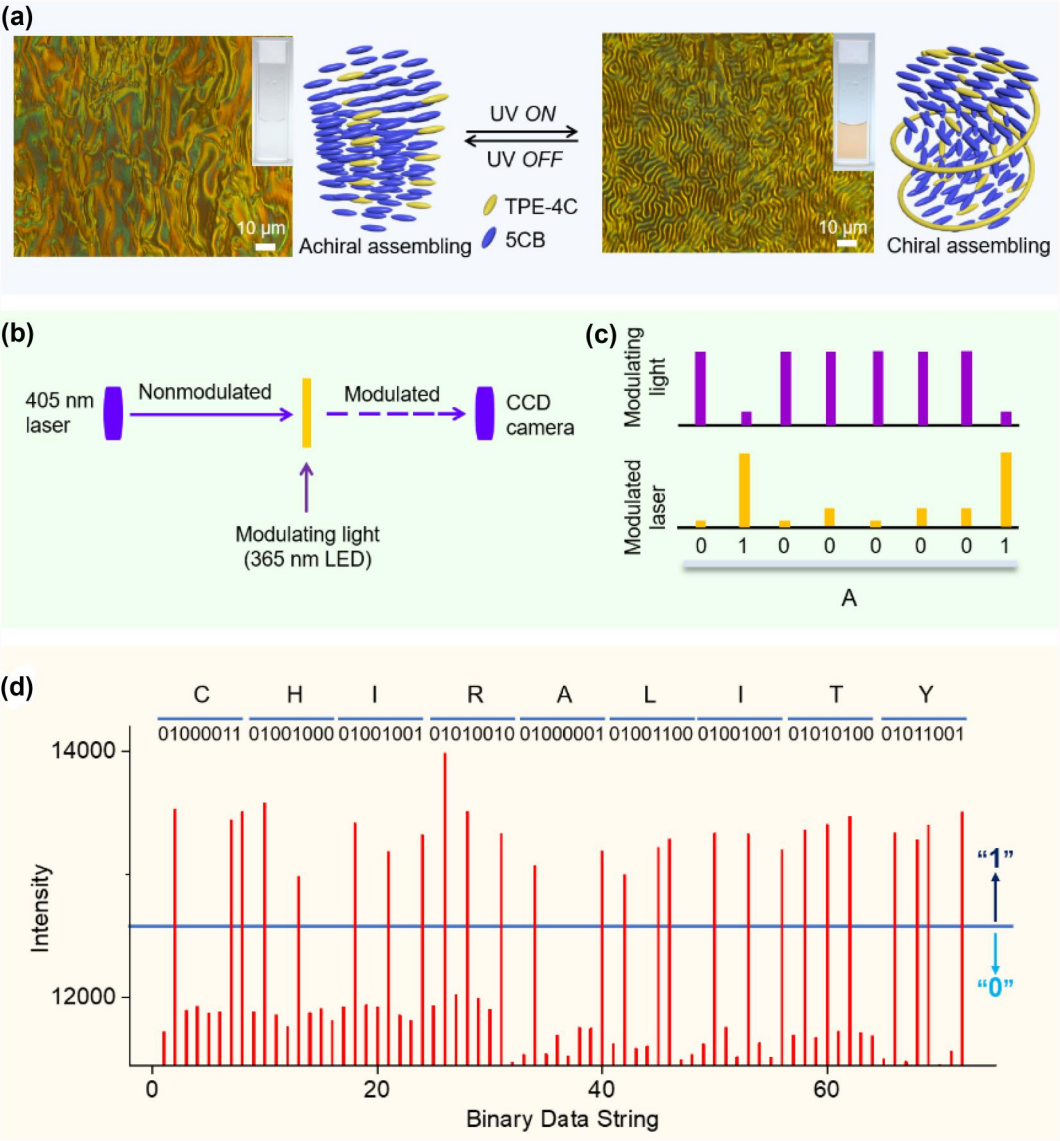
Encoding optical information in liquid crystal assembly. (a) POM images and schematic diagrams of TPE‐4C⊂5CB photoswitching between the nematic phase (schlieren texture) and cholesteric phase (fingerprint texture) before (left) and after (right) UV irradiation, respectively. Mass fraction of TPE‐4C/5CB = 3/100; temperature = 35°C. (b) The photo‐optical modulation system depicts that modulation beam (violet arrow) and the input‐output beam (405 nm laser) are 45° angle, and the sample of TPE‐4C⊂5CB was placed into microcuvette (10 × 10 × 1 mm); Input 405 nm laser is not modulated, but this laser beam is modulated after passing through an active photoswitching TPE‐4C⊂5CB under the action of an encoding light. (c) Turning on 365 nm light carries binary information of “0” and turning off 365 nm light carries binary information “1.” They impart meaningful information as follows: the modulating light (365 nm LED light) carries the information of the letter “A,” and this information is transmitted to the input 405 nm laser through the photoswitching molecules, encoding a meaningful output by detecting the intensity change of 405 nm laser. (d) The word “CHIRALITY” is encoded into the output optical fiber using the mechanism described above. Each letter has its corresponding 8‐digit binary code. POM, polarizing optical microscope; TPE, tetraphenylethene.

Considering that cholesterol is a classic LC mesogen with multiple chiral centers, we encapsulated TPE‐4C into the achiral LC matrix 5CB (Figure S1) to form an LC co‐assembly system, TPE‐4C⊂5CB. We then carried out polarized optical microscopy (POM) to explore the LC texture and gain further insight into macroscopic aspects of the co‐assembly system.[^52^, ^53^] TPE‐4C⊂5CB was found to have an achiral schlieren texture at 35°C (Figure 5a, left), demonstrating that the chiral TPE‐4C cannot induce achiral LC (5CB) into a chiral co‐assembly. Luckily, a snapshot of a chiral fingerprint texture was observed under irradiation with 365 nm light (Figure 5a, right), indicating that the cholesteric LC mesogen in *c*‐TPE‐4C can induce a chiral co‐assembly with achiral LC at 35°C. When the UV light was removed, the chiral fingerprint texture returned to its original achiral schlieren texture. During this process, the color of the LC co‐assembly was switched between colorless and yellow. From a co‐assembly perspective, this dramatic nematic/cholesteric phase transformation further confirms the photoswitching by TPE‐4C.

The dynamic fluorescence change could be observed during photoswitching of TPE‐4C⊂5CB. As illustrated in Figure S9, TPE‐4C⊂5CB emitted strongly at 505 nm, which was photoconverted to *c*‐TPE‐4C⊂5CB upon irradiation at 365 nm (100 mW cm^−2^). The resultant PSS has around 53% fluorescence decrease at 505 nm and the maximum fluorescence peak redshifts to 537 nm. Meanwhile, almost no circularly polarized luminescence (CPL) signal for TPE‐4C⊂5CB was detected before 375 nm laser irradiation. When exposed to the 375 nm laser, a right‐handed CPL signal (*λ*_max_ = 510 nm) was detected, with a luminescence dissymmetry factor of −1.6 × 10^−3^, as calculated by the equation *g*_lum_ = 2(*I*_*L*_ − *I*_*R*_)/(*I*_*L*_ + *I*_*R*_) (Figure S10). The CPL switch was ascribed to LC co‐assembly phase transformation providing a new method for monitoring TPE‐based photoswitching.

To demonstrate the striking color transformation in LC, we carried out a photo‐optical modulation experiment. In the electro‐optical modulation, an electric field applied perpendicular to a light beam travelling in a nonlinear medium imparts modulation of the output signals. Similarly, the phase transformation in the Mach–Zehnder device^[54]^ using TPE‐4C⊂5CB can also achieve the specific output modulation. As shown in Figure 5b, 365 nm LED light modulates laser intensity at 405 nm. Turning on 365 nm light switches the ring‐open TPE‐4C to the ring‐closed *c*‐TPE‐4C in 5CB, while turning off 365 nm light switches *c*‐TPE‐4C back to TPE‐4C. This automatic thermal cycloreversion is important since no need for another wavelength light source to recover its original state is beneficial to energy saving. To measure the optical signals accurately and avoid scattered light, the sample was kept at 35°C.

To illustrate this design, we transmitted alphabet letters using binary codes of standard 8‐bit ASCII characters in charge‐coupled device (CCD) spectroscopy (Figure 5b). Every letter is assigned to an 8‐digit binary code. In our experimental setting of measuring 405 nm laser intensity, turning on 365 nm light encodes “0” due to the lower optical density of 405 nm laser, resulting from the fact that *c*‐TPE‐4C could partially absorb input 405 nm laser. Conversely, turning off 365 nm light encodes “1” due to the high optical density of 405 nm laser. The encoding beam (365 nm) emerged from an optical fiber and illuminated the photoswitching sample. Figure 5b depicts a 405 nm laser beam from the left without any encoded information and passing through the modulated TPE‐4C/*c*‐TPE‐4C⊂5CB medium, the 405 nm laser is thus modulated. Compared to the other lasers at different wavelengths, such as 349, 375, 473, and 532 nm, the influence of 405 nm on the photocyclization/cycloreversion of TPE‐4C was relatively weak.

Therefore, meaningful information was encoded into different optical density lasers, and sensed by a CCD camera. Each letter is arranged in a specific sequence of eight‐digit binary codes; for example, the letter “A” consists of the data string 01000001 in binary numbers (Figure 5c). The definition of “0” is that the optical density of 405 nm laser is below 12,500 values, whereas “1” is defined when the response is greater than 12,500 values. Next, we set a data stream by switching 365 nm LED light carrying information of “CHIRALITY” (Figure 5d). In this data stream taken from 405 nm laser intensity, each letter corresponds to eight binary digits that are grouped together with a blue bar and their corresponding letter above the data. The results verify that the encoding information by the photocyclization and cycloreversion of TPE‐4C⊂5CB is transferred to the 405 nm laser without errors.

## 3 CONCLUSION

The work focuses on how to establish a visible photocyclization of TPE derivatives, thereby revealing the rationale of TPE‐based photocyclization comprehensively and exploring very insightful underlying mechanisms, such as ultrafast transient absorption spectra, reference compounds, and theoretical calculations. Thus, we have solved the issue of whether DAE‐featured TPE derivatives bestows photo‐switchable characteristics and successfully demonstrated a type of visual and reversible photocyclization of TPE by attaching cholesteryloxycarbonyloxy substituents onto the four benzene rings of TPE by increasing the energy barrier for the cycloreversion reaction. This strategy can efficiently prolong the persistence lifetime of the photocyclization state of the TPE moiety from 63 ps to 46 s by 7.20 × 10^11^‐fold, thus remarkably enhancing the thermal stability of cyclization state. The photocyclization/cycloreversion transformation can be achieved by turning on/off a UV light, along with a relatively good fatigue resistance. As observed, the color was switched from colorless to yellow upon UV irradiation, corresponding to a new absorption band in the visible region. Taking advantage of the chirality and liquid crystallinity of cholesterol, we encapsulated TPE‐4C into LC, and realized a light‐driven phase transformation between the achiral (UV *OFF*) and chiral (UV *ON*) textures in the LC co‐assembly system. This collection of desired properties enables encoding optical information, validating a photo‐optical modulation using TPE‐4C and *c*‐TPE‐4C as the bidirectional counterparts. We believe that this strategy could be valuable for further understanding of TPE, a star AIE building block, benefiting the further advancement of TPE‐based application.

## 4 EXPERIMENTAL SECTION/METHODS

*Synthesis of TPE‐*4C: 1,1,2,2‐tetrakis(4‐hydroxyphenyl)ethylene (0.5 g, 1.26 mmol), cholesteryl chloroformate (3.4 g, 7.56 mmol) and 1.0 mL pyridine were added in 50 mL dry THF under nitrogen. After 24 h reflux reaction, the mixture was cooled to room temperature, and evaporated under reduced pressure. The solid crude was washed with H_2_O and extracted with CH_2_Cl_2_. The combined organic layer was dried over anhydrous Na_2_SO_4_, and evaporated under reduced pressure. The crude product was purified by silica chromatography with hexane/CH_2_Cl_2_ (1:1 v/v), and obtained as a white solid powder TPE‐4C (1.91 g, 74% yield). ^1^H NMR (400 MHz, CDCl_3_, ppm): *δ* = 7.00 (d, *J* = 8.8 Hz, 8H, phenyl‐H), 6.94 (d, *J* = 8.8 Hz, 8H, phenyl‐H), 6.94 (d, *J* = 8.8 Hz, 8H, phenyl‐H), 5.40 (s, 4H, alkene‐H), 4.58–4.50 (m, 4H, ‐O‐C*H*(‐CH_2_‐)_2_), 2.50–2.40 (m, 8H), 2.02–0.86 (m, 152 H), 0.68 (s, 12H, ‐CH_3_). ^13^C NMR (100 MHz, CDCl_3_, ppm): *δ* = 152.82, 149.95, 140.71, 139.76, 139.29, 132.424, 123.28, 120.58, 78.96, 56.83, 56.28, 50.12, 42.46, 39.86, 39.66, 38.05, 36.98, 36.69, 36.33, 35.93, 32.06, 31.98, 28.37, 28.16, 27.76, 24.43, 23.98, 22.96, 24.43, 23.98, 22.96, 22.71, 21.20, 19.42, 18.86, 12.00. Maldi‐TOF mass spectrometry: [M + Na]^+^ calcd for [C_138_H_196_O_12_Na]: 2069.4; found 2069.4. [M + K]^+^ calcd for [C_138_H_196_O_12_K]: 2085.4; found 2085.4.

*Theoretical and computational methods*: TPE‐based isomerization on Figure 4a and Figure S3, and Table S1: The photo‐isomerization process of TPE molecules was studied by SF‐TDDFT method. All calculations were performed using the ORCA quantum chemistry software (Version 5.0.1) using the PBE0 functional and the def2‐SV(P) basis set. Grimme's D3BJ dispersion correction was used to improve the calculation accuracy. The Conductor‐like Polarization Continuum Model implicit solvation model was used to account for the solvation effect of THF.

Molecular dynamics (MD) simulation on Figure 4b,c: The MD simulation for the structural optimization of the TPE‐Chol molecule were conducted in the GROMACS 2019 software package with OPLS‐AA force field and the simulation time is 100 ns. The partial charge of the TPE‐Chol molecule was calculated using the Gaussian 16 code and the 6‐31+g(d,p) basis functions were applied. The OPLSS‐AA force field and MKTOP were used to parametrize all atoms, such as the bond parameters, angle parameters and the dihedral angles, and so on. The steepest descent method was applied to minimize the initial energy for each system with a force tolerance of 1 kJ mol^−1^ nm^−1^ and a maximum step size of 0.002 ps before MD calculations. In all the three directions, periodic boundary conditions were imposed. The leapfrog algorithm was used to integrate the Newtonian equation of motion. In constant‐pressure simulations, the pressure was maintained at 1 bar by the Berendsen barostat in an isotropic manner. The temperature was maintained by the V‐rescale thermostat at 298.15 K. The linear constraint solver algorithm was performed for constraint bond lengths of hydrogen atoms. The Particle‐Mesh‐Ewald (PME) with a fourth‐order interpolation was used to evaluate the electrostatic interactions and the grid spacing is 1.0 Å, whereas a cutoff of 1.0 Å was employed to calculate the short‐range van der Waals interactions.

Calculated absorption spectra on Figure 4d, Figures S7 and S8: The ground state geometry was optimized using MOPAC software at PM6‐D3H4 level. The excited states were calculated with linear response time‐dependent DFT (TDDFT) at the optimized ground state geometry. TDDFT calculations were performed with the Gaussian 16 package (Rev. C.01) using the hybrid B3LYP functional and the 6‐31G* basis set. Grimme's D3BJ dispersion correction was used to improve the calculation accuracy. The hole and electron distributions were performed with Multiwfn. The solvation model based on density implicit solvation mode was used to account for the solvation effect of THF.

*Analysis measurements*: The on‐line absorption spectra (Figure 3) were recorded on an Ocean Optics Maya 2000 charge‐coupled device (CCD camera), Thus we used optical density (O.D.) rather than absorbance to express the relative absorption intensity. Ultrafast transient absorption spectra were recorded on a Spectra Physics SOL ACE100F1K LP. POM images (LC texture) were recorded on a Leica Dmlp.

## CONFLICT OF INTEREST STATEMENT

The authors declare no conflicts of interest.

## ETHICS STATEMENT

No animal or human experiments were involved in this study.

## Supporting information

Supporting Information S1

## Data Availability

The data that support the findings of this study are available in the supplementary material of this article.
