# Supplementary material for: Enhancing tetraphenylethene cyclization as photoswitch
Source: Smart Mol. 2023 Aug 6;1(2):e20230003. doi: 10.1002/smo.20230003 (PMC12118202; doi:10.1002/smo.20230003)
Supplement: Supplementary file 1 — Supporting Information S1 [file SMO2-1-e20230003-s001.docx]

Supporting Information

# Enhancing Tetraphenylethene Cyclization as Photoswitch

Yue Wu,*^1^ Yiran Ren,^1^ Xiaoxuan Zeng,^1^ Honglong Hu,^2^ Mengqi Li,^2^ Junzi Li,^3^ Tingchao He,^3^ Xin-Shun Li,^1^ Zhen-Qiang Yu,*^1^ and Wei-Hong Zhu*^2^

^1^College of Chemistry and Environmental Engineering, Center for AIE Research, Shenzhen University, Shenzhen 518071, China

^2^Laboratory for Advanced Materials and Joint International Research Laboratory of Precision Chemistry and Molecular Engineering, Feringa Nobel Prize Scientist Joint Research Center, Shanghai Key Laboratory of Functional Materials Chemistry, Institute of Fine Chemicals, Frontiers Science Center for Materiobiology and Dynamic Chemistry, School of Chemistry and Molecular Engineering, East China University of Science and Technology, Shanghai 200237, China

^3^Key Laboratory of Optoelectronic Devices and Systems of Ministry of Education and Guangdong Province, College of Physics and Optoelectronic Engineering, Shenzhen University, Shenzhen 518060, China

*Corresponding Authors: [wuyue@szu.edu.cn](mailto:wuyue@szu.edu.cn); [zqyu@szu.edu.cn](mailto:zqyu@szu.edu.cn); [whzhu@ecust.edu.cn](mailto:whzhu@ecust.edu.cn)

**Catalogs**

**1. Theoretical calculation details**

**2. Synthetic routes of TPE-4C, TPE-4P, TPE-4Bu**

**3.** **Properties of Tetraphenylethene Cyclization and calculation simulation**

**4.** **Characterization Spectra of Chemical Structures**

**1. Theoretical calculation details**

**TPE-based isomerization** on Figure 4a and Figure S3, and Table S1: The photo-isomerization process of TPE molecules was studied by SF-TDDFT (Spin-flip time-dependent density functional theory) method. All calculations were performed with the ORCA quantum chemistry software (Version 5.0.1)^1^ using the PBE0 functional and the def2-SV(P) basis set.^2^ Grimme's D3BJ dispersion correction^3^ was used to improve calculation accuracy. The CPCM (Conductor-like Continuum Polarization Model) implicit solvation model^4^ was used to account for the solvation effect of THF.

**Molecular dynamics simulation** on Figure 4b and 4c: The molecular dynamics (MD) simulation for the structural optimization of the TPE-Chol molecule were conducted in the GROMACS 2019 software package^5-9^ with OPLS-AA force field^10^ and the simulation time is 100 ns. The partial charge of TPE-Chol molecule was calculated using Gaussian 16 code^11^ and the 6-31+g(d,p) basis functions were applied.^12^ The OPLSS-AA force field^10^ and MKTOP^13^ were used to parametrize all atoms, such as the bond parameters, angle parameters and the dihedral angles, and so on. The steepest descent method was applied to minimize the initial energy for each system with a force tolerance of 1 kJ/(mol^−1^ nm^−1^) and a maximum step size of 0.002 ps before MD calculations.^14^ In all the three directions, periodic boundary conditions were imposed. Leapfrog algorithm was used to integrate the Newtonian equation of motion.^14^ In NPT simulations, the pressure was maintained at 1 bar by the Berendsen barostat in an isotropic manner.^15^ The temperature was maintained by the V-rescale thermostat at 298.15 K. The LINCS algorithm^16^ was performed for constrain bond lengths of hydrogen atoms. The Particle-Mesh-Ewald (PME) with a fourth-order interpolation was used to evaluate the electrostatic interactions and the grid spacing is 1.0 Å,^17^ whereas a cutoff of 1.0 Å was employed to calculate the short-range van der Waals interactions.

**Calculated absorption spectra** on Figure 4d, Figure S7 and Figure S8: The ground state geometry was optimized using MOPAC software at PM6-D3H4 level.^18^ The excited states were calculated with linear response time-dependent DFT (TDDFT) at the optimized ground state geometry. TDDFT calculations were performed with the Gaussian 16 package (Rev. C.01)^11^ using the hybrid B3LYP functional1^9,20^ and the 6-31G* basis set. Grimme's D3BJ dispersion correction^3^ was used to improve calculation accuracy. The hole and electron distributions were perforemed with Multiwfn.^21^ The SMD implicit solvation mode^22^ was used to account for the solvation effect of THF.

**References**

F. Neese, *WIREs Comput. Mol. Sci.* **2022**, *12*, e1606.

F. Weigend, R. Ahlrichs, *Phys. Chem. Chem. Phys.* **2005**, *7*, 3297.

S. Grimme, S. Ehrlich, L. Goerigk, *J. Comp. Chem.* **2011**, *32*, 1456.

1. V. Barone, M. Cossi, *J. Phys. Chem. A* **1998**, *102*, 1995.
2. D. V. D. Spoel, E. Lindahl, B. Hess, et al. *J. Comput. Chem.* **2005**, *26*, 1701.
3. M. J. Abraham, T. Murtola, R. Schulz, S. Páll, J. C. Smith, B. Hess, E. Lindahl, *SoftwareX* **2015**, *6*, 19.
4. S. Páll, M. J. Abraham, C. Kutzner, B. Hess, E. Lindahl, Tackling exascale software challenges in molecular dynamics simulations with GROMACS, Solving Software Challenges for Exascale. 8759 (2015) 3–27.
5. S. Pronk, S. Páll, R. Schulz, P. Larsson, *Bioinformatics* **2013**, *29*, 845.
6. B. Hess, C. Kutzner, D. van der Spoel, E. Lindahl, *J. Chem. Theory. Comput.* **2008**, *4*, 435.
7. W. L. Jorgensen, D. S. Maxwell, J. Tirado-Rives. *J. Am. Chem. Soc.* **1996**, *118*, 11225.
8. Gaussian 16, Revision C.01, M. J. Frisch, G. W. Trucks, H. B. Schlegel, G. E. Scuseria, M. A. Robb, J. R. Cheeseman, G. Scalmani, V. Barone, G. A. Petersson, H. Nakatsuji, X. Li, M. Caricato, A. V. Marenich, J. Bloino, B. G. Janesko, R. Gomperts, B. Mennucci, H. P. Hratchian, J. V. Ortiz, A. F. Izmaylov, J. L. Sonnenberg, D. Williams-Young, F. Ding, F. Lipparini, F. Egidi, J. Goings, B. Peng, A. Petrone, T. Henderson, D. Ranasinghe, V. G. Zakrzewski, J. Gao, N. Rega, G. Zheng, W. Liang, M. Hada, M. Ehara, K. Toyota, R. Fukuda, J. Hasegawa, M. Ishida, T. Nakajima, Y. Honda, O. Kitao, H. Nakai, T. Vreven, K. Throssell, J. A. Montgomery, Jr., J. E. Peralta, F. Ogliaro, M. J. Bearpark, J. J. Heyd, E. N. Brothers, K. N. Kudin, V. N. Staroverov, T. A. Keith, R. Kobayashi, J. Normand, K. Raghavachari, A. P. Rendell, J. C. Burant, S. S. Iyengar, J. Tomasi, M. Cossi, J. M. Millam, M. Klene, C. Adamo, R. Cammi, J. W. Ochterski, R. L. Martin, K. Morokuma, O. Farkas, J. B. Foresman, and D. J. Fox, Gaussian, Inc., Wallingford CT, 2019.
9. G. A. Petersson, M. A. Al-Laham, *J. Chem. Phys.* **1991**, *94*, 6081.
10. A. A. S. T. Ribeiro, B. A. C. Horta, R. B. de Alencastro, *J. Braz. Chem. Soc.* **2008**, *19*, 1433.
11. W. F. Van Gunsteren, H. Berendsen, *Mol. Simul.* **1988**, *1*, 173.
12. H. J. C. Berendsen, J. P. M. Postma, W. F. van Gunsteren, A. DiNola, J. R. Haak, *J. Chem. Phys.* **1984**. *81*, 3684.
13. B. Hess, H. Bekker, H. J. Berendsen, J. G. Fraaije, *J. Comput. Chem.* **1997**, *18*, 1463.
14. T. Darden, D. York, L. Pedersen, *J. Chem. Phys.* **1993**, *98*, 10089.
15. J. Řezáč, P. Hobza, *J. Chem. Theory Comput.* **2012**, *8*, 141.
16. *Density-functional theory of atoms and molecules.* R.G. Parr, W. Yang, Oxford University Press, New York, Oxford, 1995.

K. Burke, *J. Chem. Phys.* **2012**, *136*, 150901.

T. Lu, F. Chen, *J. Comput. Chem.***2012**, *33*, 580.

1. A. V. Marenich, C. J. Cramer, D. G. Truhlar, *J. Phys. Chem.* B **2009**, *113*, 6378.

**2. Synthetic routes of TPE-4C, TPE-4P, TPE-4Bu**

**TPE-4C**

1,1,2,2-tetrakis(4-hydroxyphenyl)ethylene (0.5 g, 1.26 mmol), cholesteryl chloroformate (3.4 g, 7.56 mmol) and 1.0 mL pyridine were added in 50 mL dry THF under nitrogen. After 24 h reflux reaction, the mixture was cooled to room temperature, and evaporated under reduced pressure. The solid crude was washed with H_2_O and extracted with CH_2_Cl_2_. The combined organic layer was dried over anhydrous Na_2_SO_4_, and evaporated under reduced pressure. The crude product was purified by silica chromatography with hexane/CH_2_Cl_2_ (1:1 v/v), and obtained as a white solid powder TPE-4C (1.91 g, 74% yield). ^1^H NMR (400 MHz, CDCl_3_, ppm): *δ* = 7.00 (d, *J* = 8.8 Hz, 8H, phenyl-H), 6.94 (d, *J* = 8.8 Hz, 8H, phenyl-H), 6.94 (d, *J* = 8.8 Hz, 8H, phenyl-H), 5.40 (s, 4H, alkene-H), 4.58-4.50 (m, 4H, -O-C*H*(-CH_2_-)_2_), 2.50–2.40 (m, 8H), 2.02–0.86 (m, 152 H), 0.68 (s, 12H, -CH_3_). ^13^C NMR (100 MHz, CDCl_3_, ppm): *δ* = 152.82, 149.95, 140.71, 139.76, 139.29, 132.424, 123.28, 120.58, 78.96, 56.83, 56.28, 50.12, 42.46, 39.86, 39.66, 38.05, 36.98, 36.69, 36.33, 35.93, 32.06, 31.98, 28.37, 28.16, 27.76, 24.43, 23.98, 22.96, 24.43, 23.98, 22.96, 22.71, 21.20, 19.42, 18.86, 12.00. Maldi-TOF mass spectrometry: [M + Na]^+^ calcd for [C_138_H_196_O_12_Na]: 2069.4; found 2069.4. [M + K]^+^ calcd for [C_138_H_196_O_12_K]: 2085.4; found 2085.4.

**TPE-4P**

Compound TPE-4P was synthesized according to TPE-4C with 77% yield. ^1^H NMR (400 MHz, CDCl_3_, ppm): *δ* = 7.05–7.02 (m, 8H, phenyl-H), 6.99–6.95 (m, 8H, phenyl-H), 4.21 (t, *J* = 6.7 Hz, 8H, -O-CH_2_-), 1.78 (m, 8H, -CH_2_-), 1.02 (t, *J* = 7.4 Hz, 12H, -CH_3_). ^13^C NMR (100 MHz, CDCl_3_, ppm): *δ* =153.49, 149.84, 140.64, 139.65, 132.33, 120.46, 70.34, 21.97, 10.19. ESI mass spectrometry: [M + H]^+^ calcd for [C_42_H_45_O_12_]: 741. 2906; found 741.2907.

**TPE-4Bu**

Under an N_2_ atmosphere, a two-necked flask equipped with a magnetic stirrer was charged with zinc powder (244.26 mg, 3.74 mmol) and 20 mL THF. The mixture was cooled to -5 to 0 °C, and TiCl_4_ (0.21 mL, 1.87 mmol) was slowly added by a syringe with the temperature kept under 10 °C. The suspending mixture was warmed to room temperature and stirred for 0.5 h, then heated at reflux for 2.5 h. The mixture was again cooled to -5 to 0 °C, charged with pyridine (0.15 mL, 1.87 mmol) and stirred for 20 min. The solution of bis(4-(tert-butyl)phenyl)methanone (1.0 g, 1.70 mmol) in 10 mL THF was added slowly. After addition, the reaction mixture was heated at reflux until the carbonyl compounds were consumed (monitored by TLC). The reaction was quenched with 5% HCl aqueous solution and filter the catalyst with diatomaceous earth loading column. The organic filtrate was collected and concentrated. The crude product was purified by silica chromatography with hexane/CH_2_Cl_2_ (10:1 v/v), and obtained as a white solid powder TPE-4Bu (133.1 mg, 12% yield). ^1^H NMR (400 MHz, CDCl_3_, ppm): *δ* = 7.07–7.05 (d, *J* = 8.4 Hz, 8H, phenyl-H), 6.93–6.91 (d, *J* = 8.4 Hz, 8H, phenyl-H), 1.24 (s, 36H, -CH_3_). ^13^C NMR (100 MHz, CDCl_3_, ppm): *δ* = 148.83, 141.08, 140.25, 130.92, 124.16, 34.35, 31.29. ESI mass spectrometry: [M + H]^+^ calcd for [C_42_H_53_]: 557.4142; found 557.4141.

**TPE-4OMe, TPE-4A, and TPE-4TPA**

TPE-4OMe, TPE-4A, and TPE-4TPA in Figure S5 are commercially available from Bide Pharmatech.

**3. Properties of Tetraphenylethene Cyclization and calculation simulation**

Figure S1. Chemical structure of liquid crystal 5CB.


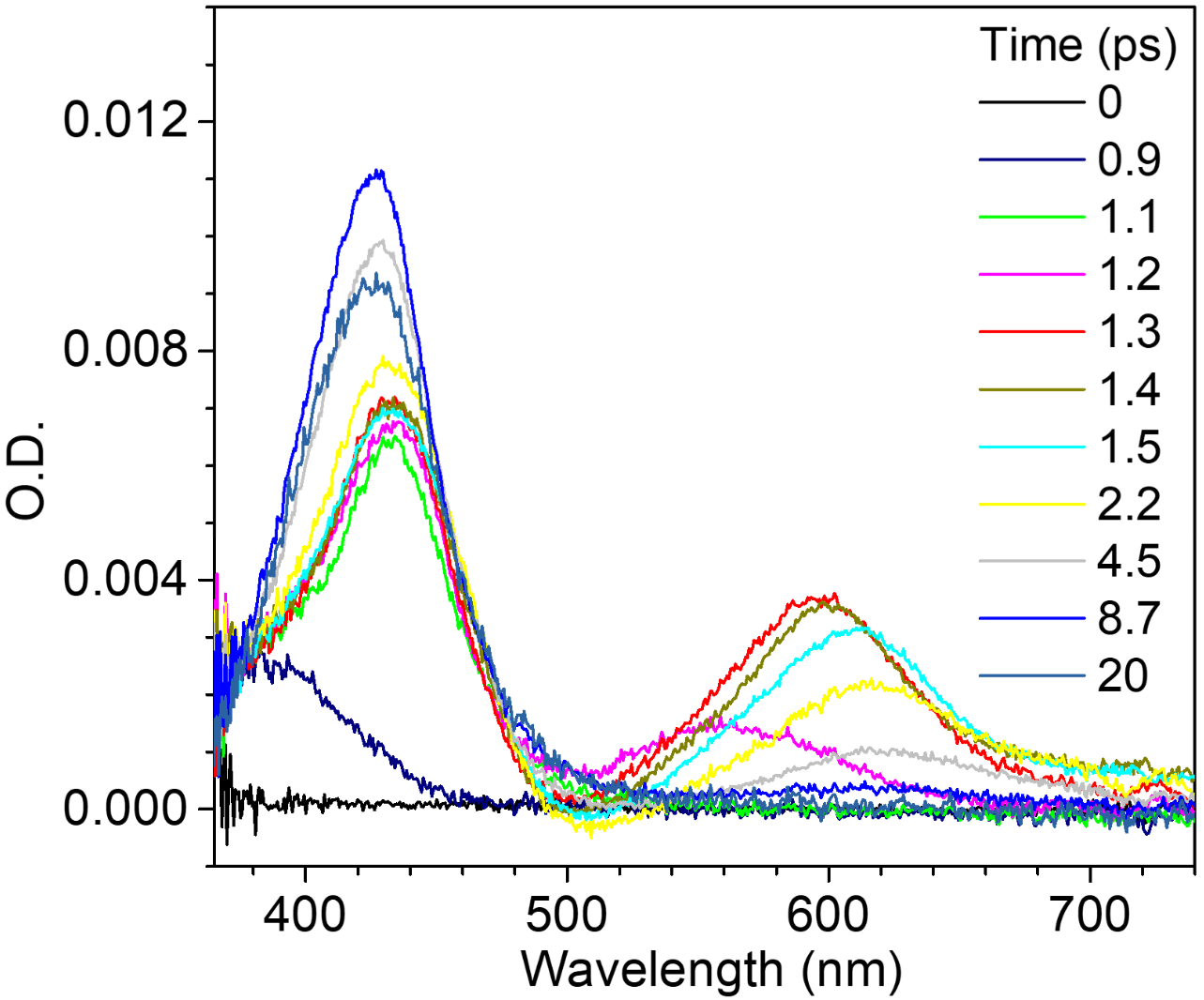


Figure S2. Transient absorption spectra of TPE monitored at 0, 0.9, 1.1, 1.2, 1.3, 1.4, 1.5, 2.2, 4.5, 8.7, and 20 ps, respectively.


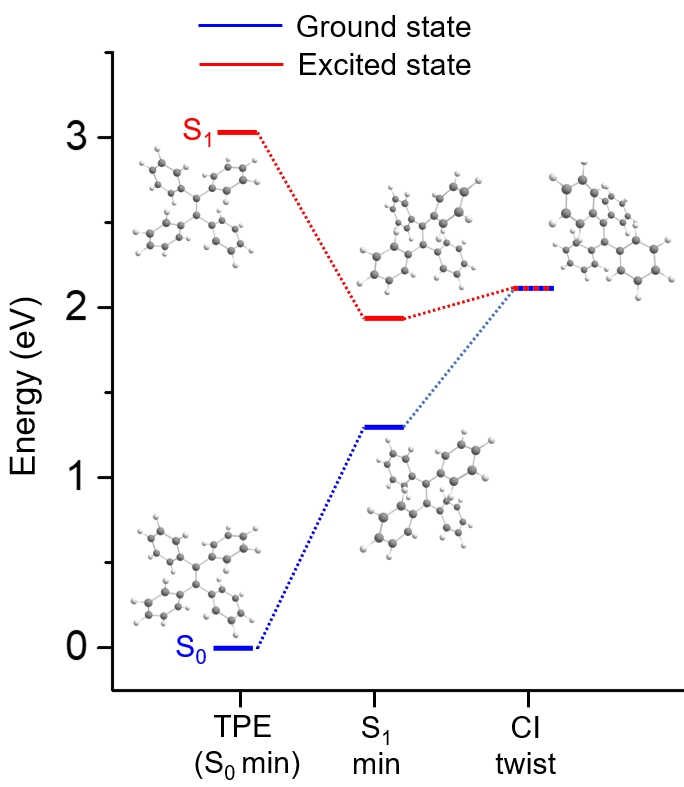


Figure S3. Computed energy profile of TPE twisting after electronic excitation. The excited TPE molecule can deactivate by the *cis-trans* isomerization by twisting around the central ethylene bond.

Table S1. Calculated energy level of optimized TPE and TPE-4P in the different states


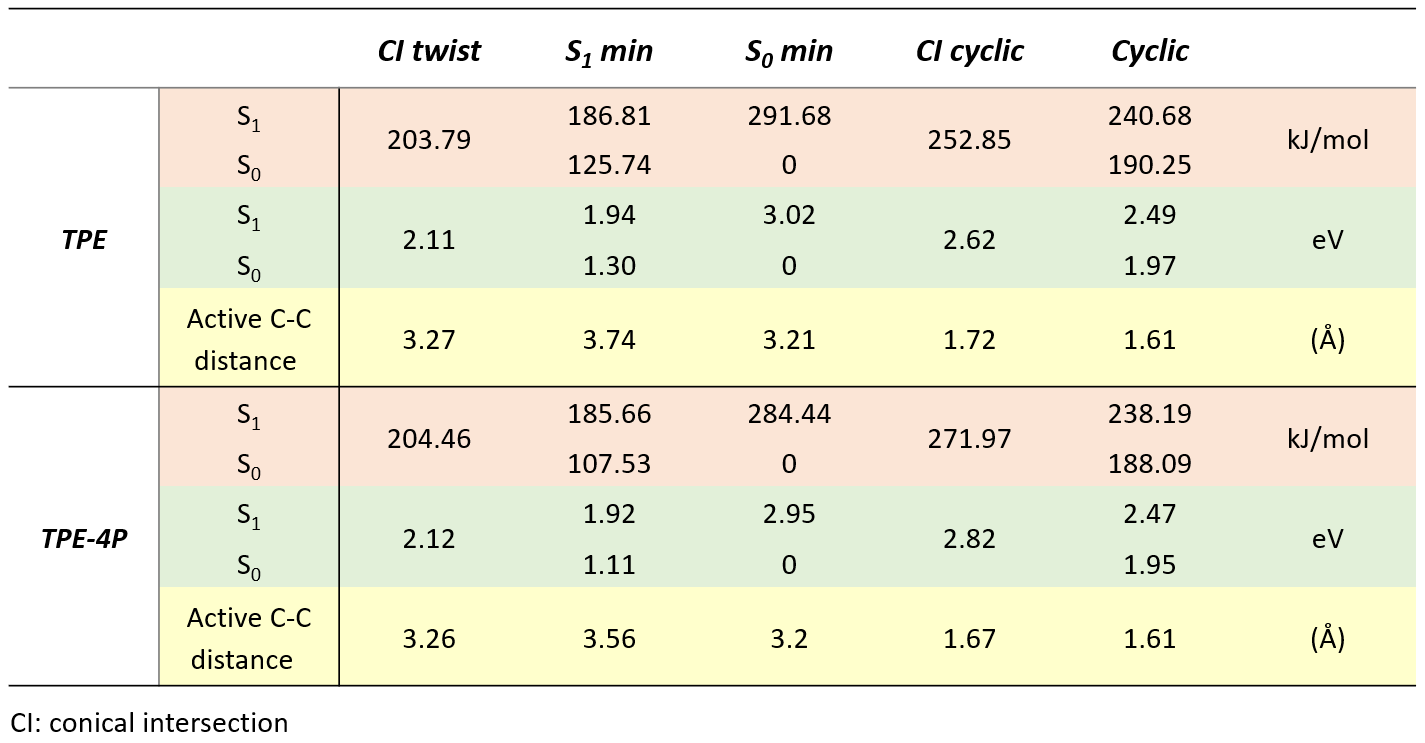


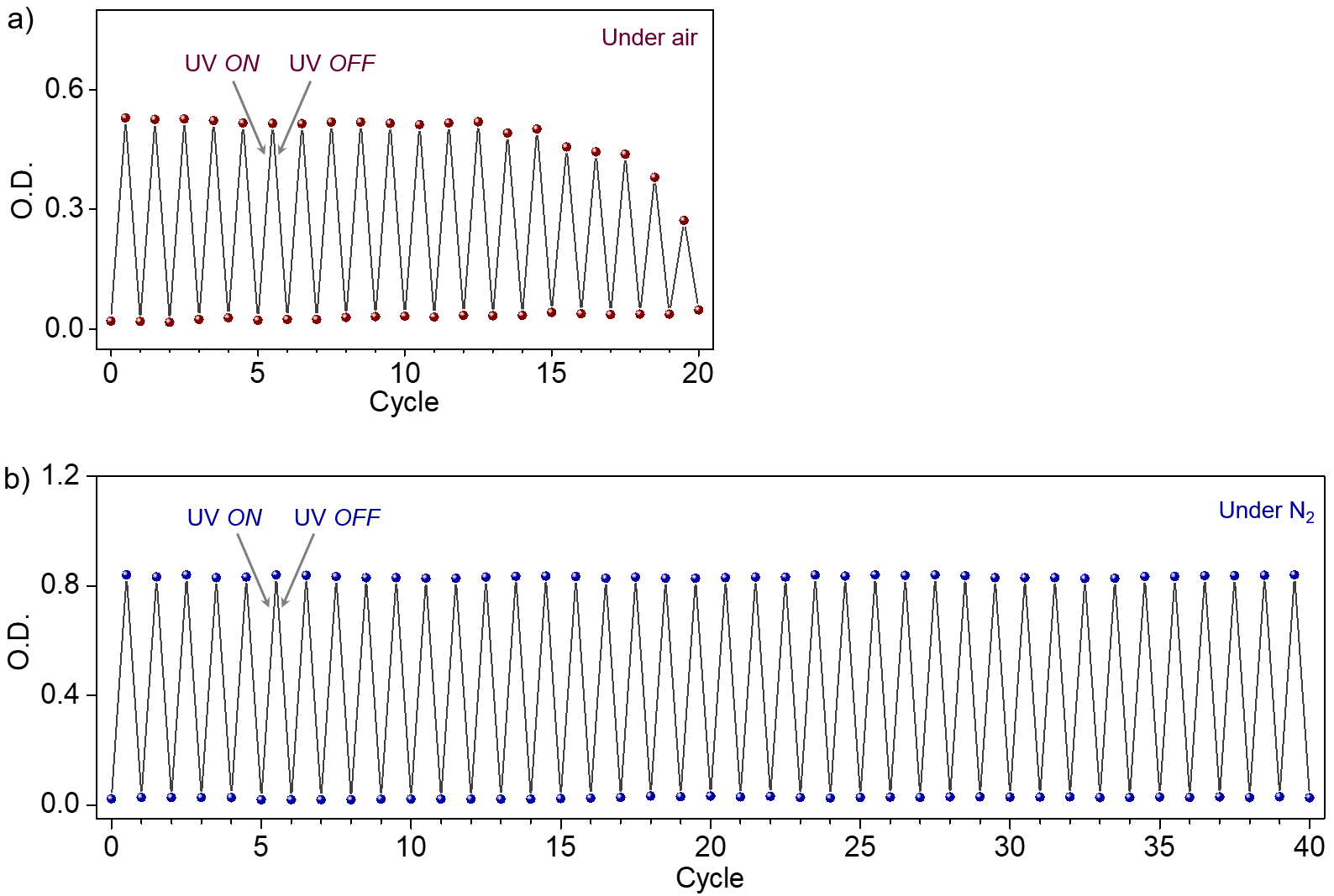


Figure S4. On-off toggling of photoswitch between ring-open and ring-closed forms of TPE-4C by alternating between UV (365 nm, 100 mW cm^-2^) on and off under air (a) or N_2_ (b) environment, respectively.


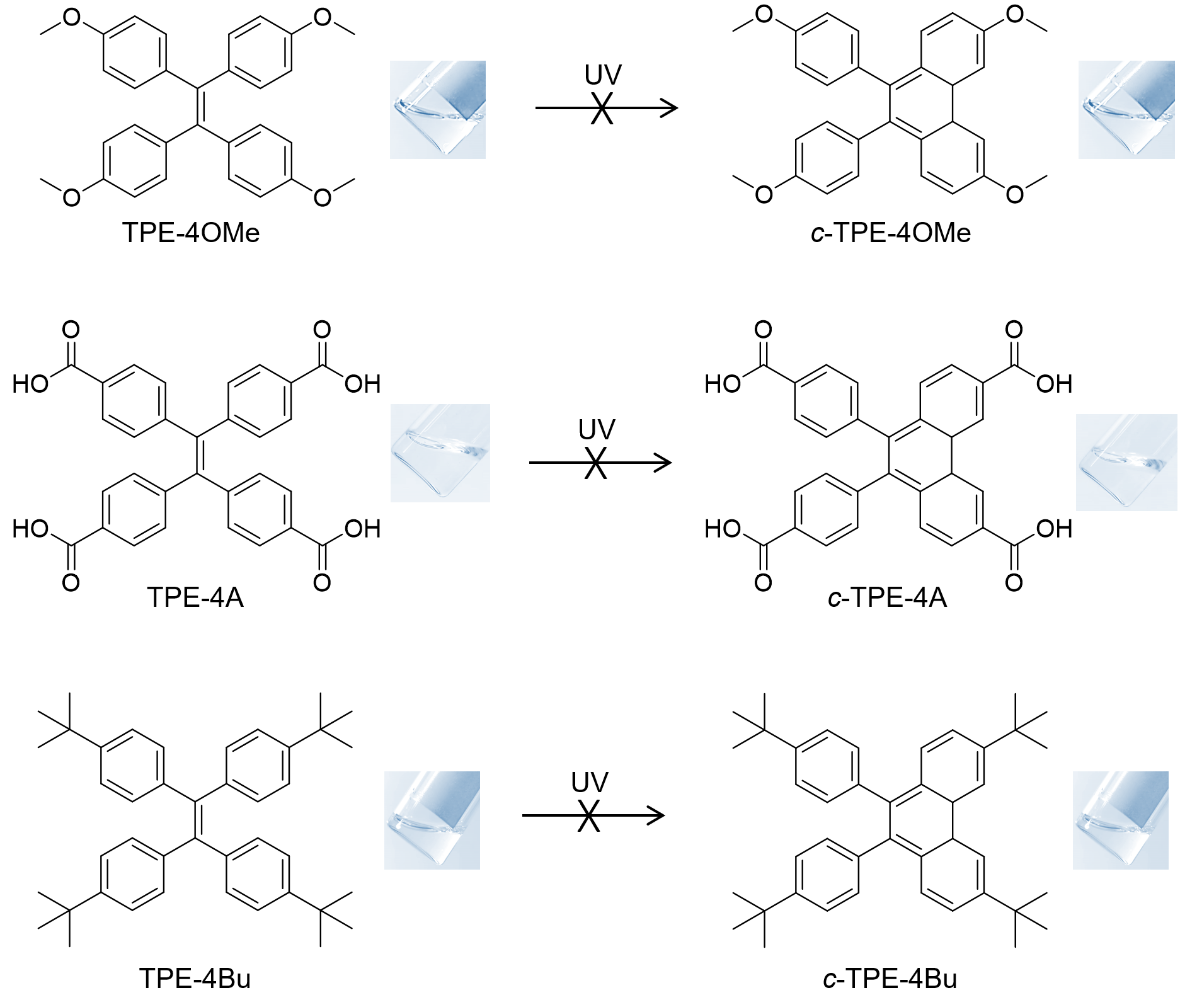


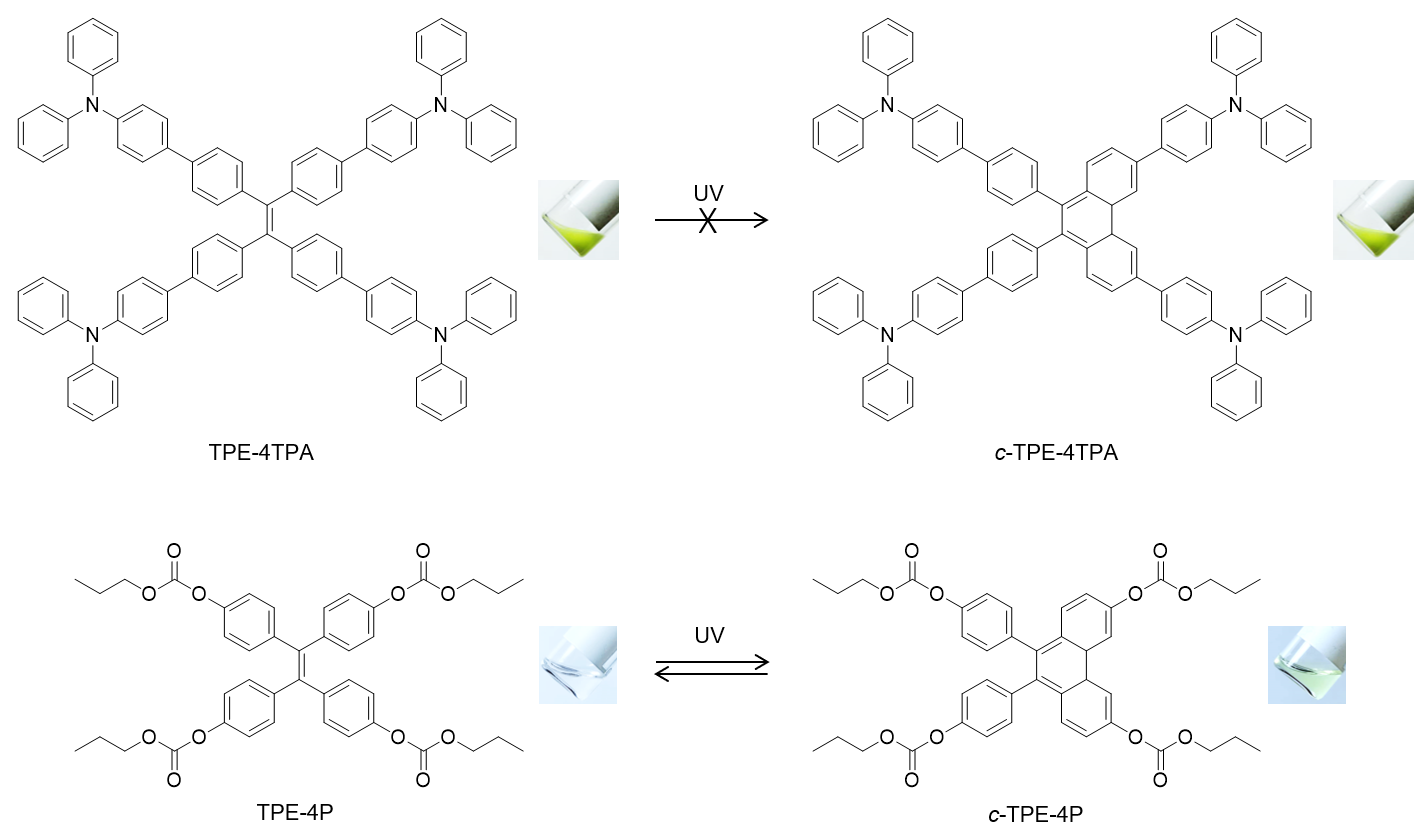


Figure S5. Similar to TPE, upon irradiation with UV light, diarylethene structures TPE-4OMe, TPE-4A, TPE-4Bu, and TPE-4TPA are incapable of visible photocyclization. Similar to TPE-4C, TPE-4P, with four carbonate esters, does display photocyclization monitored by the naked eyes.


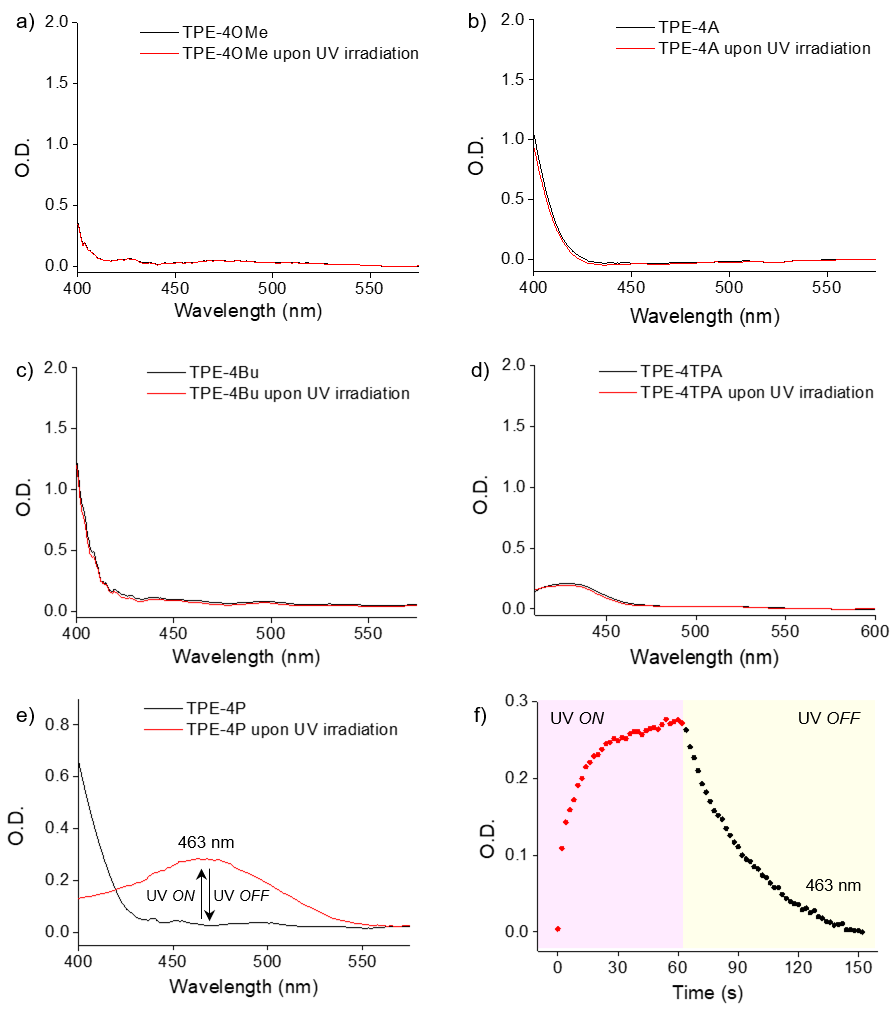


Figure S6. Absorption spectra of TPE-4OMe (a), TPE-4A (b), TPE-4Bu (c), TPE-4TPA (d), and TPE-4P (e) before and upon 365 nm irradiation in THF (10 mM). (f) Photocyclization and cycloreversion kinetics of TPE-4P measured at 463 nm as a function of UV on and off time.


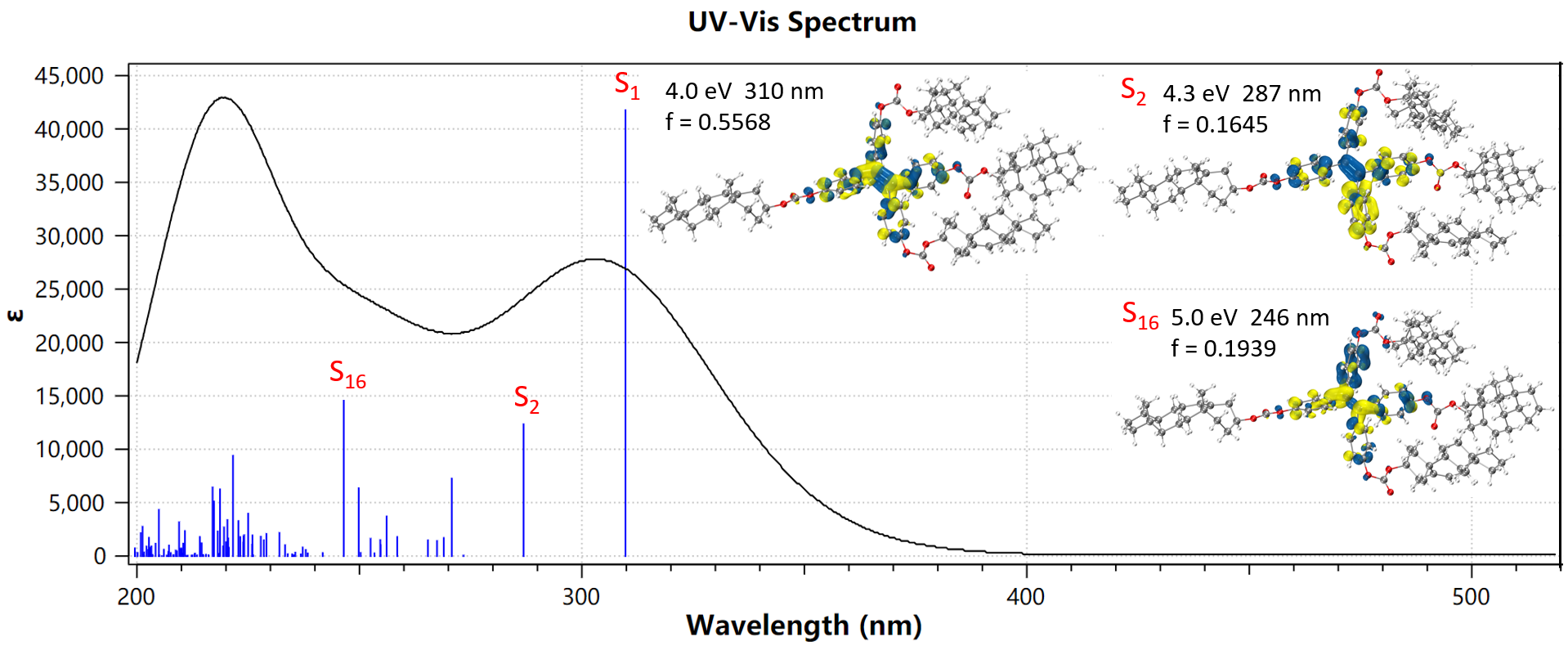


Figure S7. Calculated absorption spectrum and dominating hole and electron distributions of ring-open form (TPE-4C, black line) in THF.


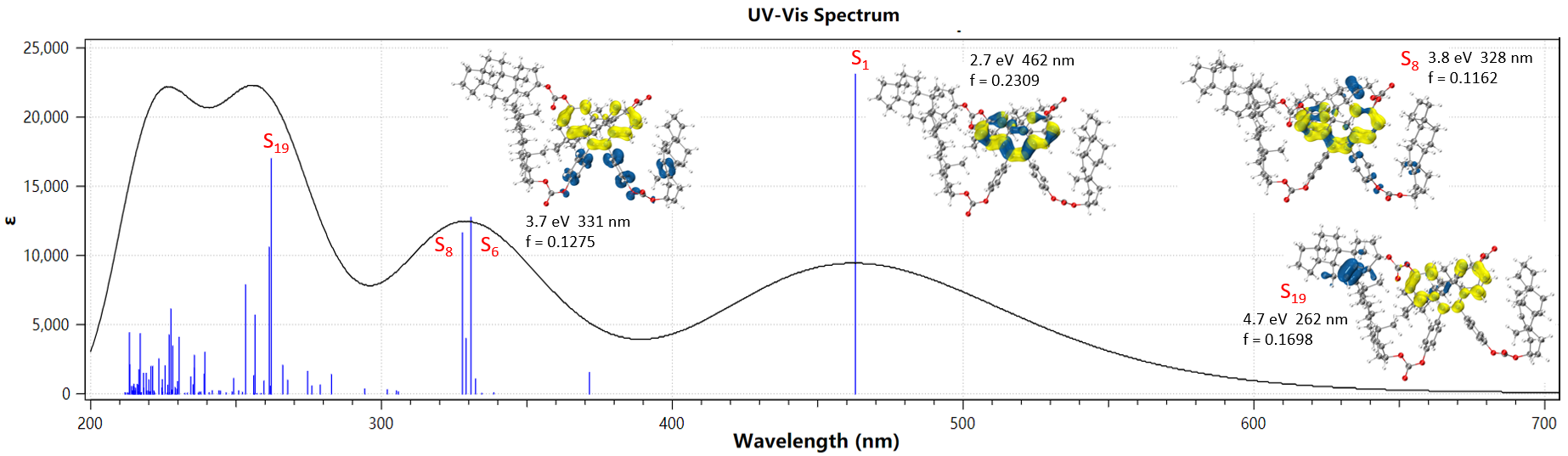


Figure S8. Calculated absorption spectrum and dominating hole and electron distributions of ring-closed form (*c*-TPE-4C) in THF.


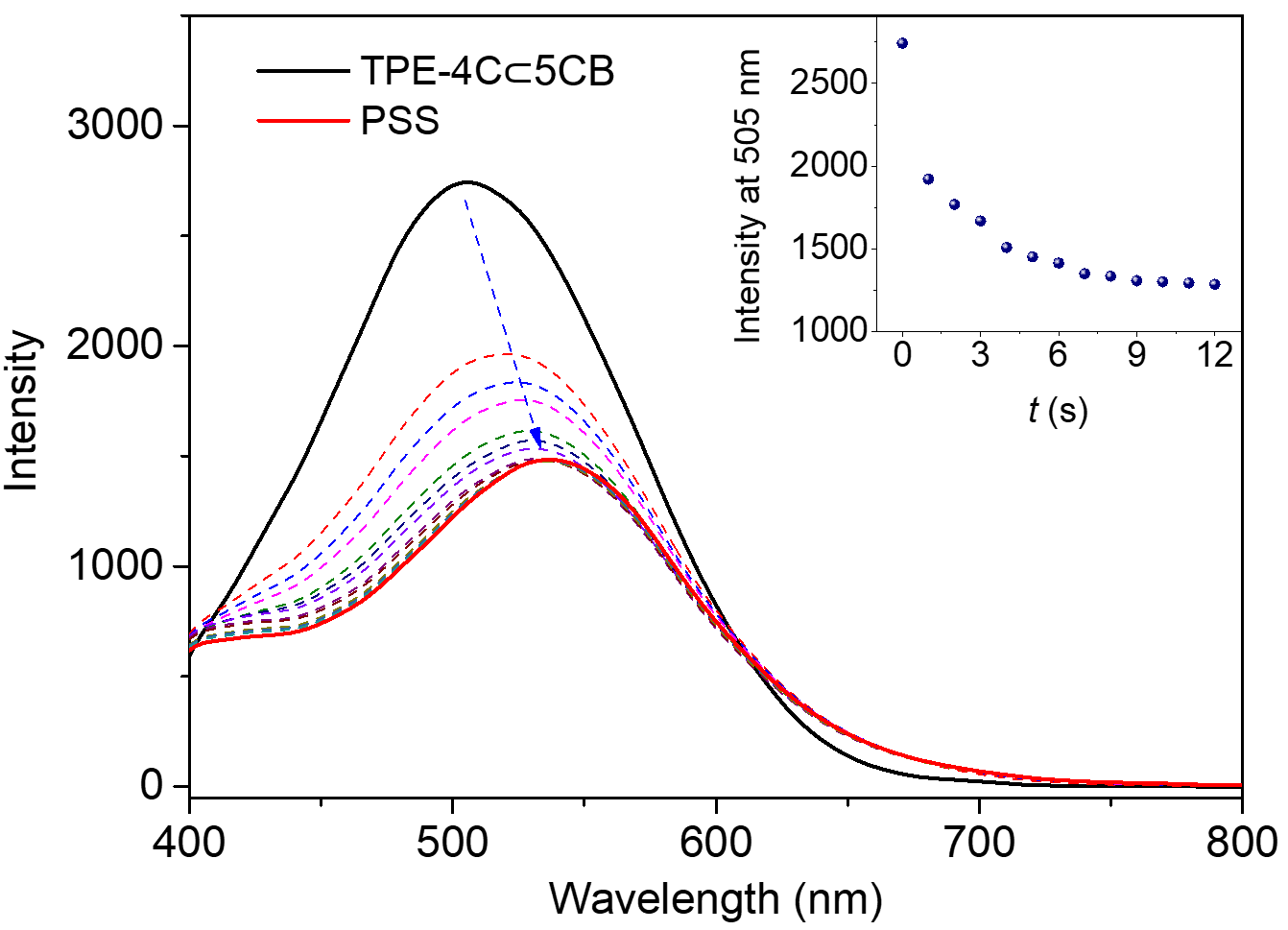


Figure S9. Fluorescence changeof TPE-4C⊂5CB (TPE-4C / 5CB = 0.5% w/w) upon 365 nm light irradiation. Insets: fluorescence intensity changes at 505 nm. PSS: photostationary state.


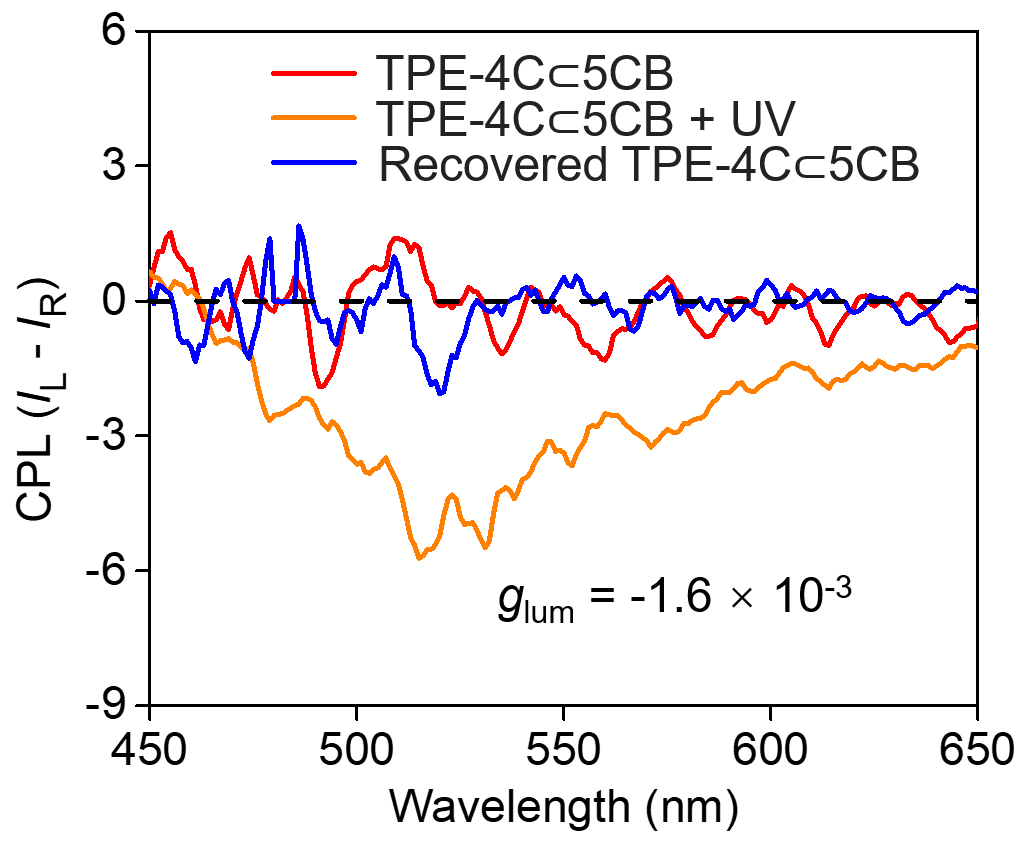


Figure S10. CPL of TPE-4C⊂5CB (red line) before UV irradiation and PSS (orange line) upon 375 nm laser irradiation (20 mW/cm^2^), and then switch off 375 nm laser, the CPL signal convert back to silent CPL (blue line). Notes: TPE-4C / 5CB = 0.5% w/w; optical path of cuvette = 1 mm.

**4. Characterization Spectra of Chemical Structures**


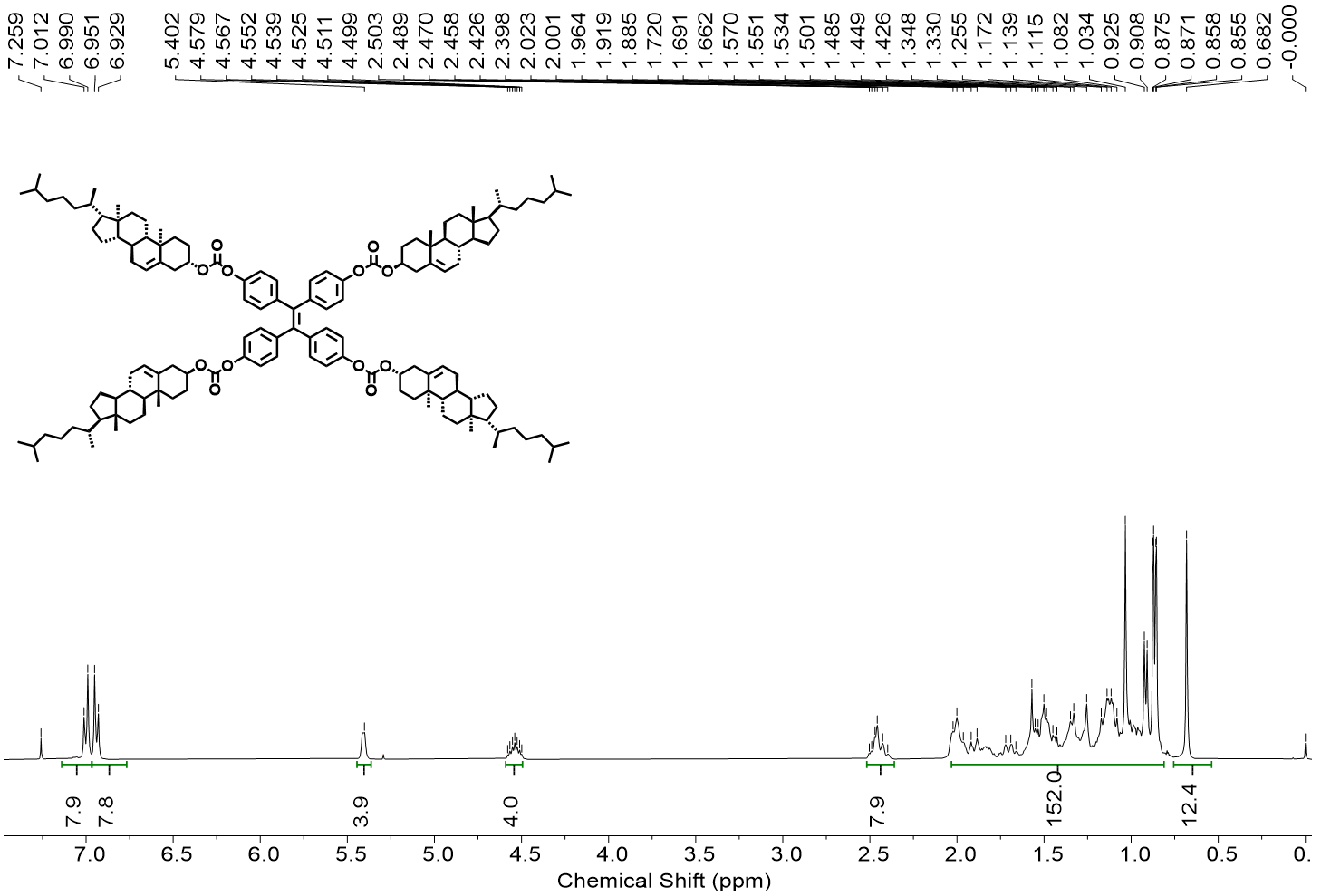


Figure S11. ^1^H NMR of TPE-4C in CDCl_3_.


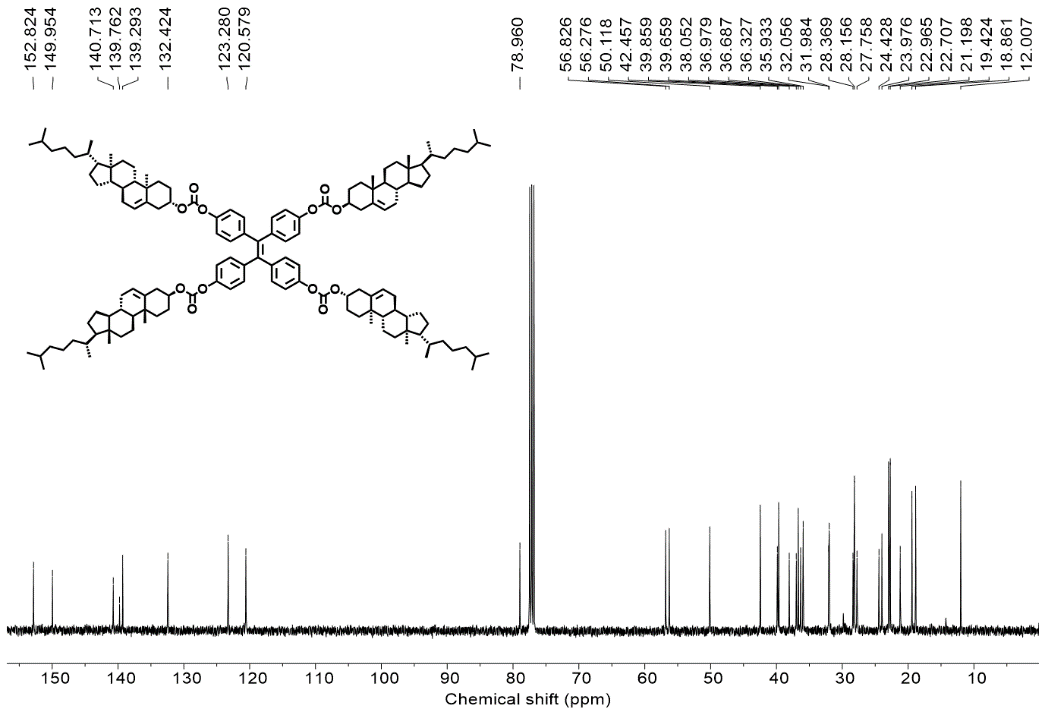


Figure S12. ^13^C NMR of TPE-4C in CDCl_3_.


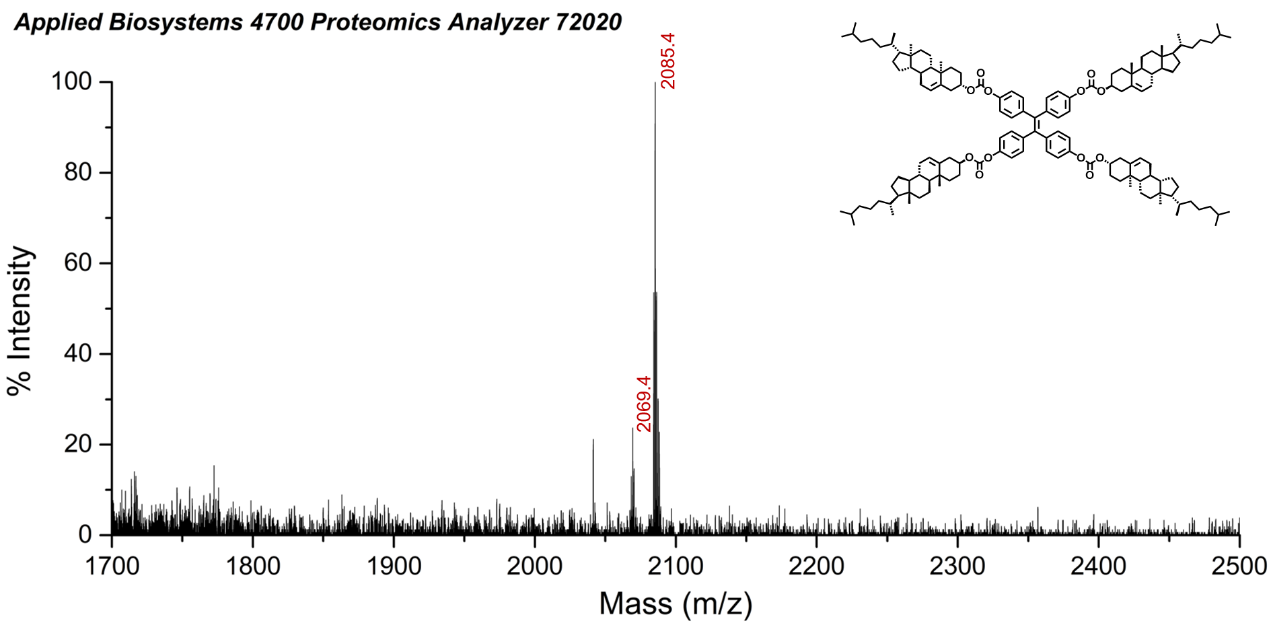


Figure S13. Maldi-TOF mass spectrum of TPE-4C.


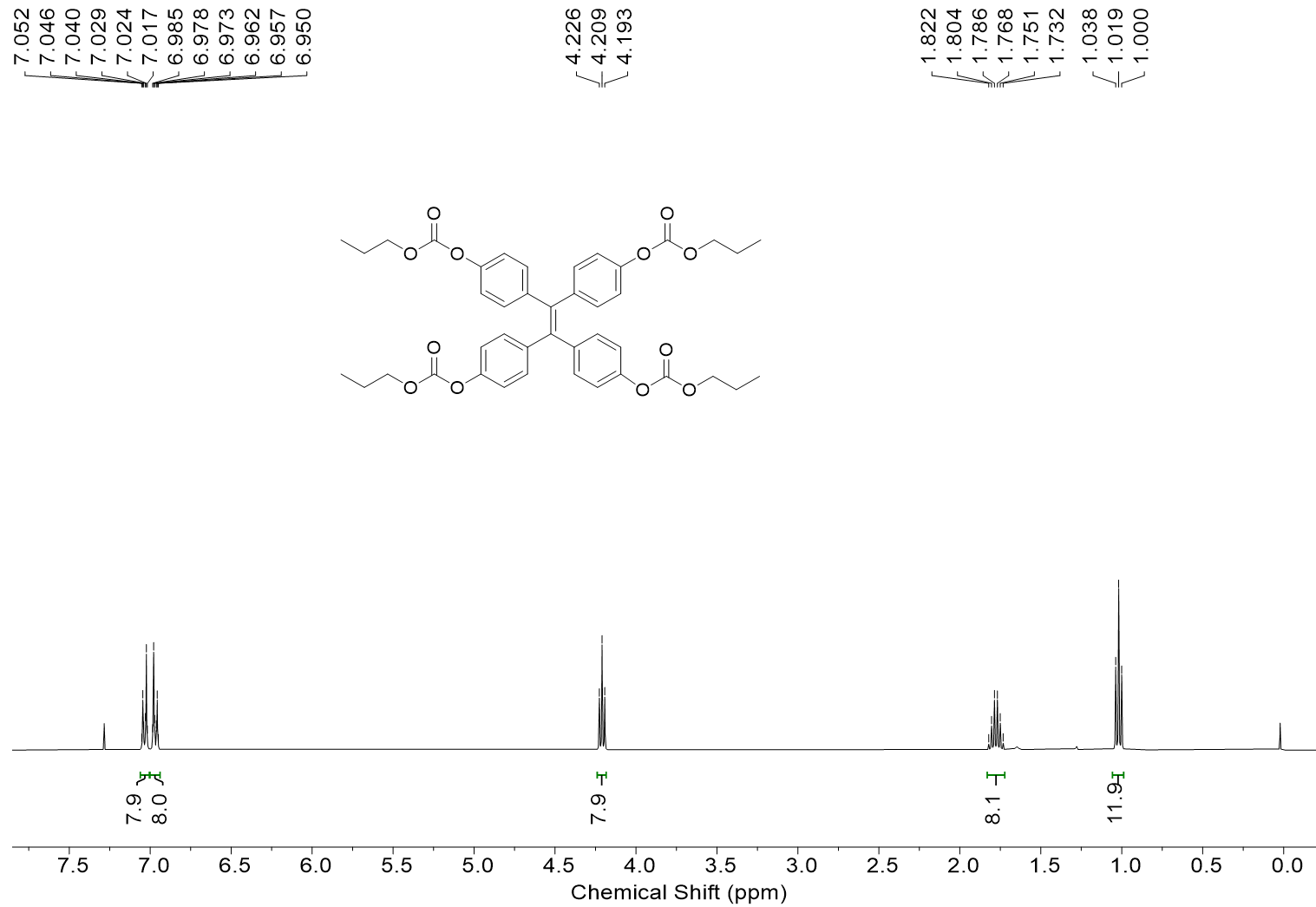


Figure S14. ^1^H NMR of TPE-4P in CDCl_3_.


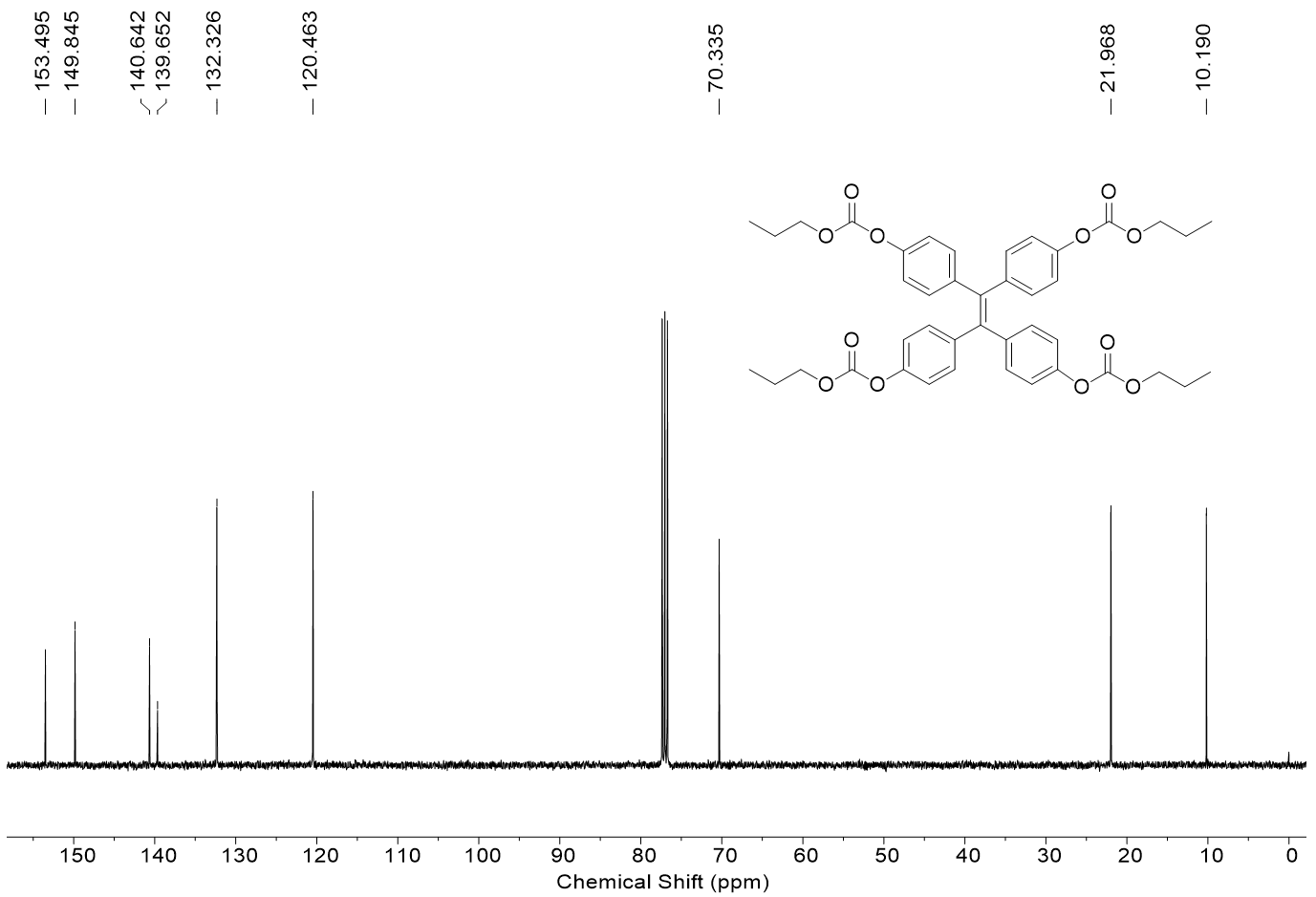


Figure S15. ^13^C NMR of TPE-4P in CDCl_3_.


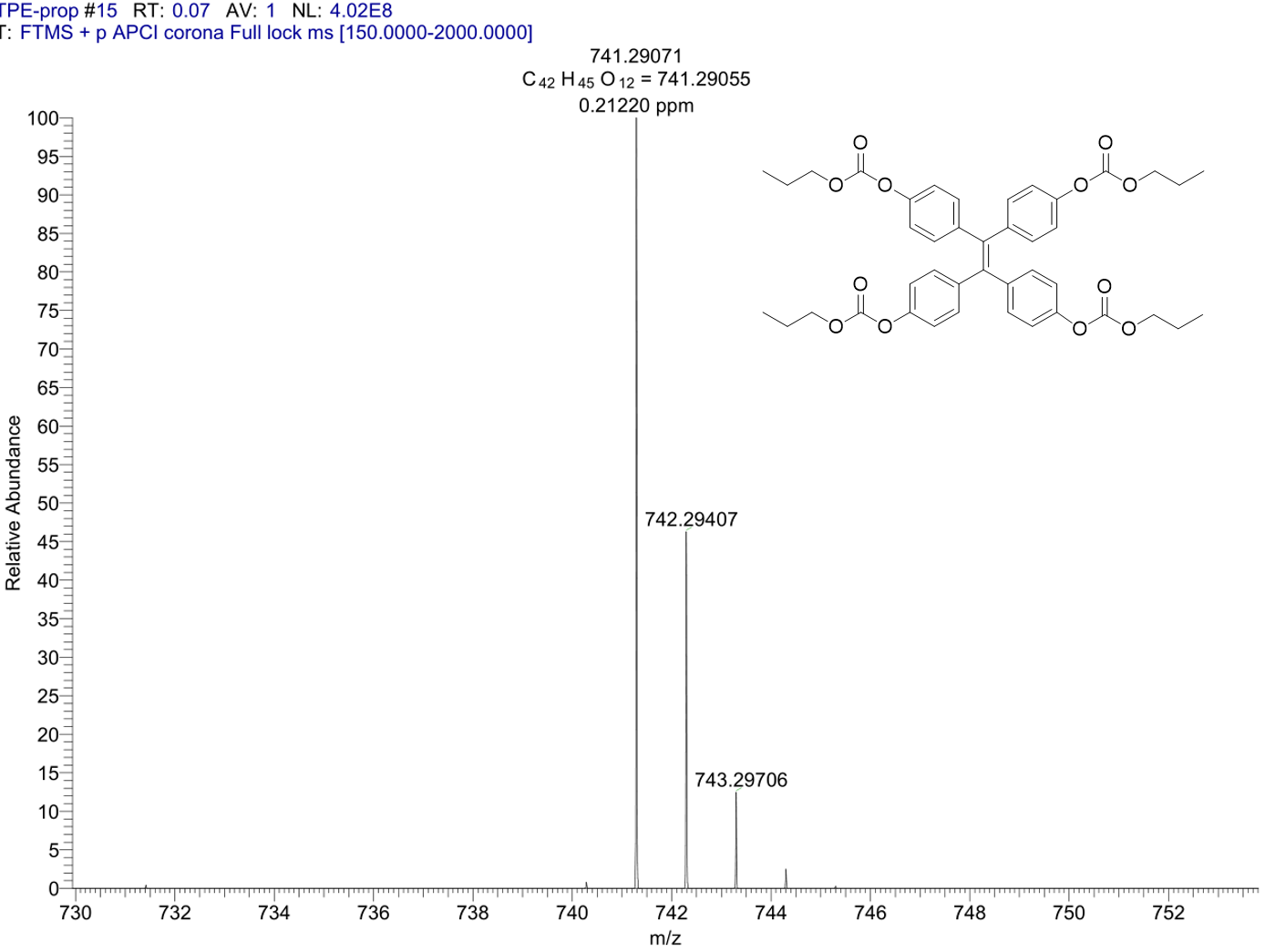


Figure S16. ESI mass spectrum of TPE-4P.


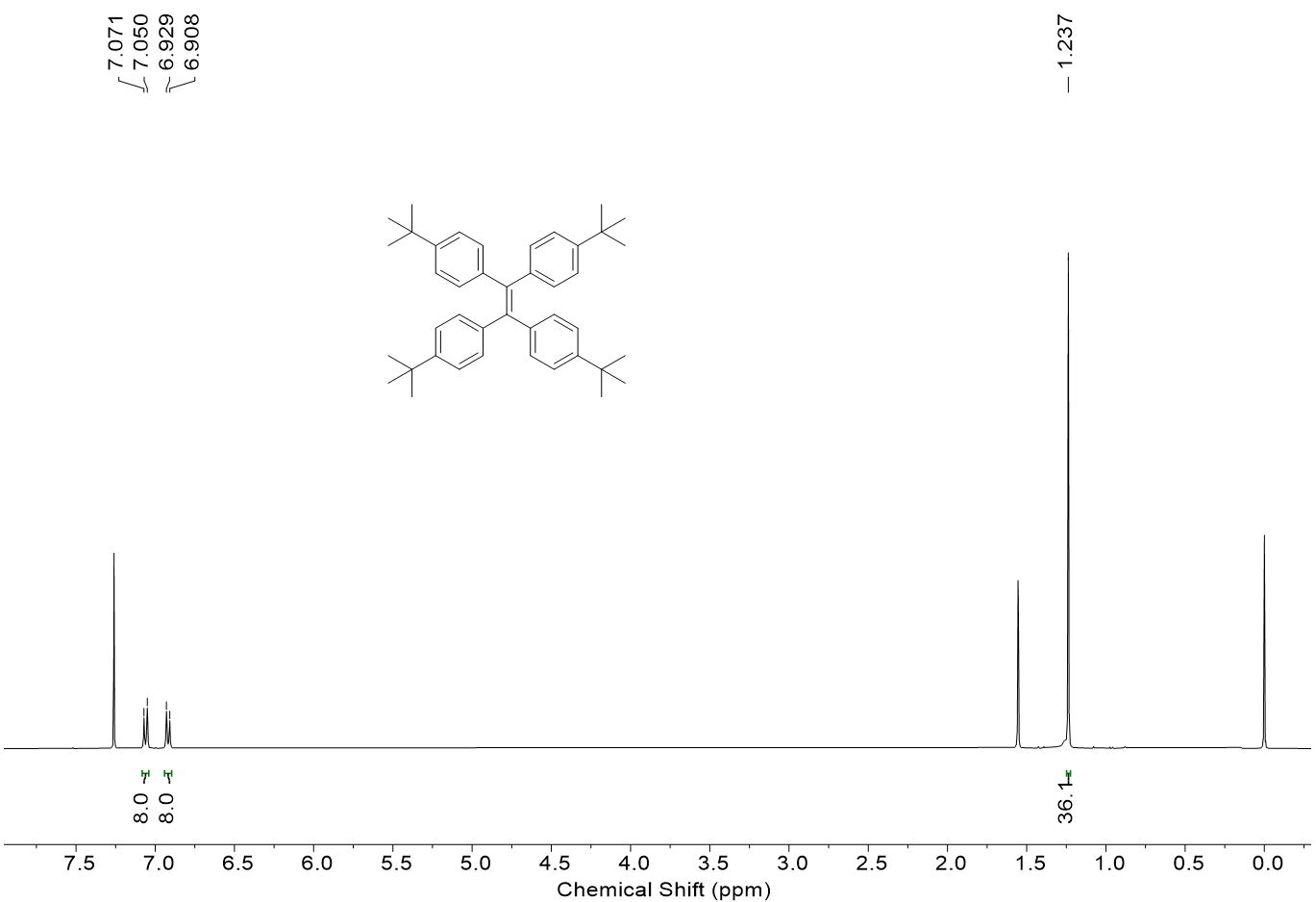


Figure S17. ^1^H NMR of TPE-4Bu in CDCl_3_.


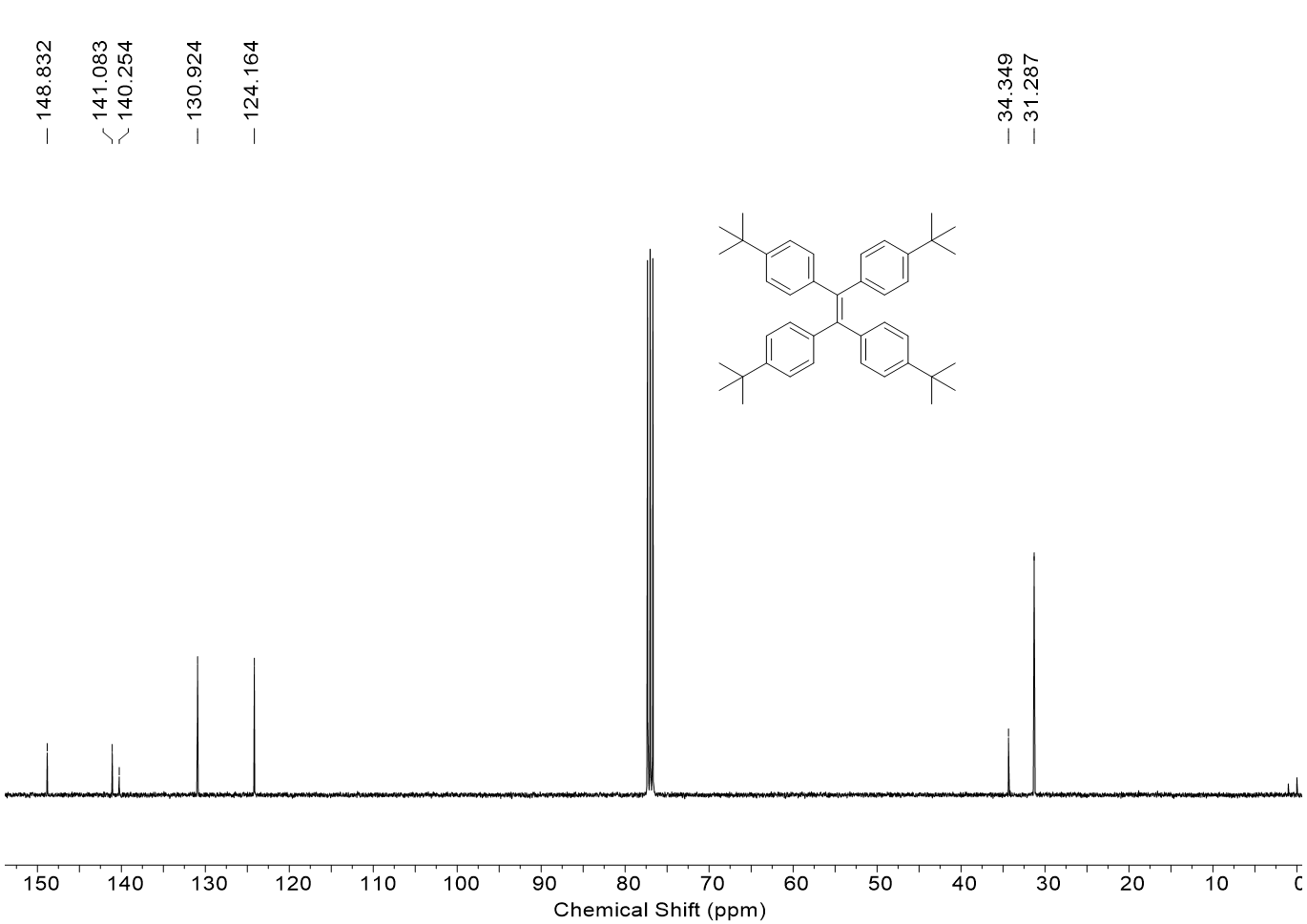


Figure S18. ^13^C NMR of TPE-4Bu in CDCl_3_.


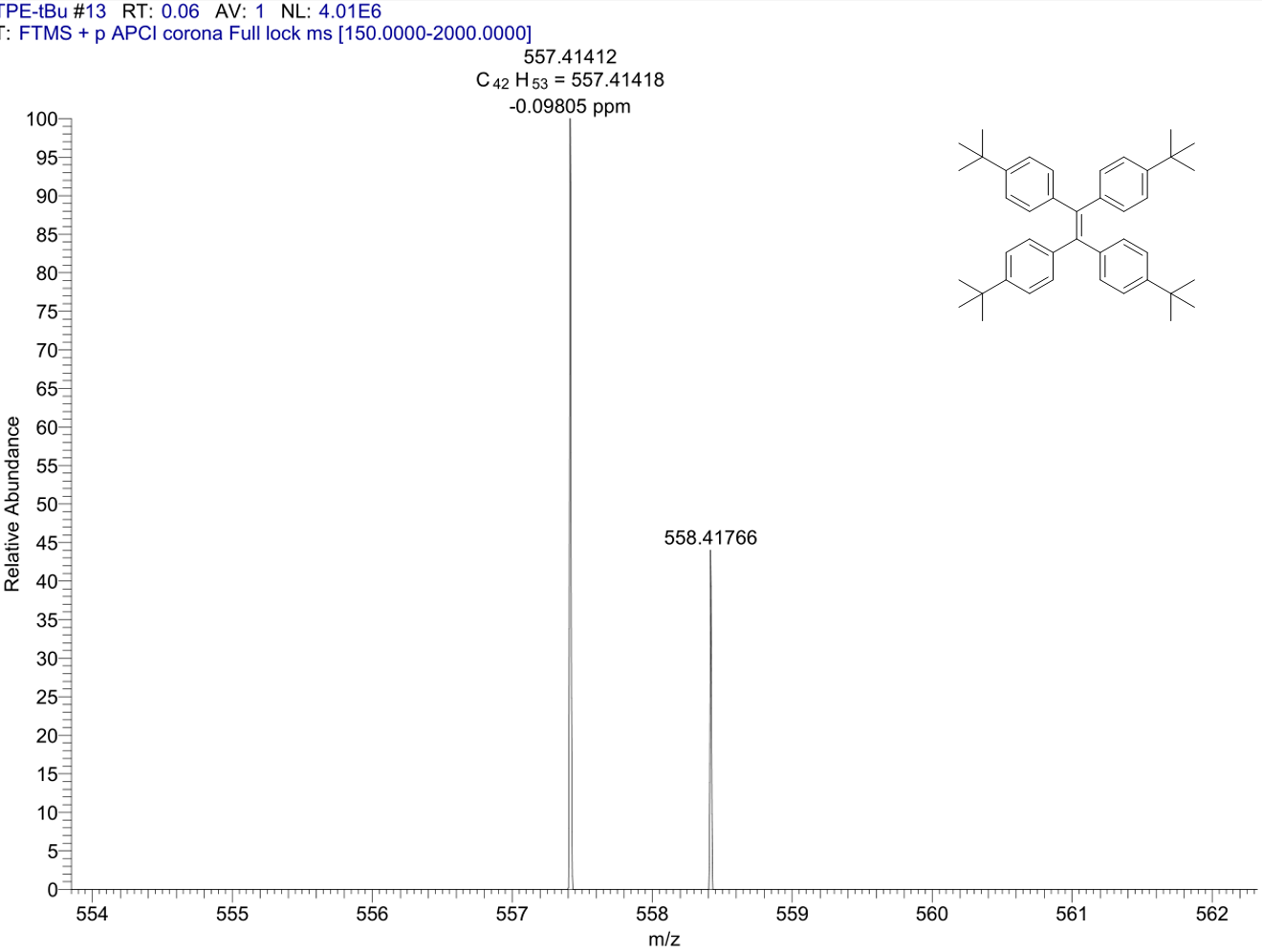


Figure S19. ESI mass spectrum of TPE-4Bu.
